# Long-Term Labeling and Differentiation Monitoring of Mouse Spermatogonial Stem Cells Using CellREADR

**DOI:** 10.3390/biotech15040077

**Published:** 2026-09-08

**Authors:** Shuaitao Hu, Lin Yan, Gulistan Khan, Xiaowei Liu, Chunsheng Han

**Affiliations:** 1 State Key Laboratory of Organ Regeneration and Reconstruction, Institute of Zoology, Chinese Academy of Sciences, Beijing 100101, China; hushuaitao22@ioz.ac.cn (S.H.); yanlin@ioz.ac.cn (L.Y.); gulistankhan741@gmail.com (G.K.); lxw_0825@163.com (X.L.); 2 Institute for Stem Cell and Regeneration, Chinese Academy of Sciences, Beijing 100101, China; 3 Beijing Institute for Stem Cell and Regenerative Medicine, Beijing 100101, China; 4 University of Chinese Academy of Sciences, Beijing 100049, China; 5 Kunming Institute of Zoology, Chinese Academy of Sciences, Kunming 650204, China

**Keywords:** live-cell labeling, RNA sensing, cell type identification, spermatogonial stem cells

## Abstract

Cellular identity determination and lineage tracing is a pivotal technique in modern biological research. Conceptually simple yet efficient cell labeling techniques offering broader applicability are warranted despite many methods requiring intricate construction procedures. One such technique is ADAR (adenosine deaminase acting on RNA)-mediated RNA sensing, a live cell labeling technique that is based on the expression and abundance of cell-type-specific RNAs. Here, we utilized the optimized version, CellREADR (Cell access through RNA sensing by Endogenous ADAR), to establish a feasible tracing system for mouse spermatogonial stem cells (mSSCs) which are refractory to CRISPR-based reporter gene knock-in. We identified several previously unreported features of CellREADR, including RNA interference induced by double-strand RNA formation that is obligatorily generated during the normal operation of the CellREADR system. More importantly, we established its application for long-term labeling of mSSCs and monitoring mSSC differentiation induced by retinoic acid (RA) treatment in vitro. This work offers a practical solution for dynamic monitoring of mSSC self-renewal and differentiation and supports that CellREADR can be developed into more versatile and efficient tools in stem cell research.

## 1. Introduction

The diverse cell types and their specialized functions in a multicellular organism are determined by the expression products of the same genome including transcripts, proteins, and epigenetic modifications [1,2]. Accurate discrimination and tracing of cellular identity based on their intrinsic molecular profiles represent an important aspect of modern cell biology. Contemporary methods are mainly based on the direct detection of gene products using RT-PCR, immunostaining, or various omics technologies. Although these methods provide greater accuracy than methods based on morphological descriptions [3], they usually compromise cell integrity and viability [4,5,6,7]. In contrast, live-cell labeling technologies offer a promising alternative by enabling long-term, real-time monitoring of cells.

Live-cell labeling relies on the engineering of gene expression circuits, resulting in novel physical or chemical features that allow cells to be monitored noninvasively [8,9,10]. The most commonly used labeling technology involves employing the CRISPR/Cas9 system to insert reporter genes into specified genome loci, thereby reflecting endogenous gene expression through the activity of reporter genes to indicate cell states. However, this approach is hindered by several limitations including low success rates, time-consuming procedures, and the potential risk for the inserted reporter gene to disrupt the function of endogenous genes [11,12,13]. MicroRNA expression levels vary substantially across different cell types [14], prompting the development of a microRNA-based live-cell labeling strategy—the microRNA switch—for monitoring cellular identity transitions and isolating specific cell populations derived from induced pluripotent stem cells (iPSCs) [15,16,17]. However, this approach is still limited by challenges such as high background noise.

**Figure 1:**
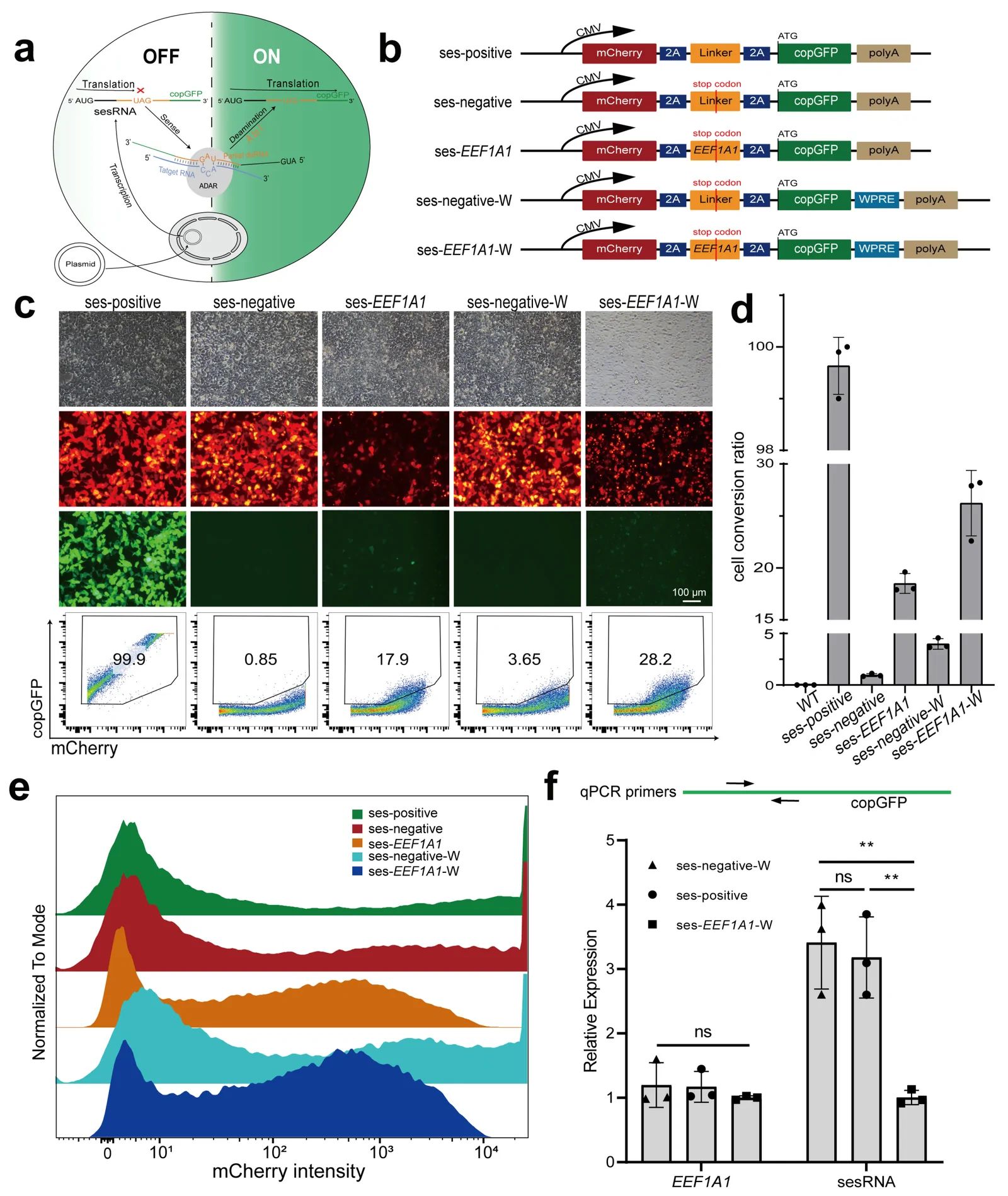
Further characterization of CellREADR. (**a**) Schematic diagram of the RNA sensing technology principle. After the plasmid is transfected into cells then transcription. If there is a target RNA in the cell that can form a double-stranded RNA with sesRNA, the RNA sensing system is activated, leading to the expression of the effector gene. By contrast, translation of the effector signal does not occur. (**b**) Schematic diagram of the plasmid structure for testing the effectiveness of the RNA sensing system and the WPRE. In the ses-positive and ses-negative groups, a short linker is used to express RNA that cannot target endogenous RNA within the cell. The red vertical lines indicate that a stop codon has been artificially introduced to create a mismatch. The “-W” indicates that the WPRE has been additionally added to the original plasmid structure. (**c**) Brightfield, fluorescence of image and flow cytometry results for each group after plasmid transfer into cells; scale bar is 100 μm. (**d**) The positive rate of each group was calculated according to the results of flow cytometry. (**e**) The fluorescence intensity of the reporter mCherry was compared across the experimental groups corresponding to the plasmid vectors presented in (**a**). (**f**) The primer pairs targeting copGFP for the sesRNA plasmid are shown. RT-qPCR was used to quantify the relative expression levels of endogenous EEF1A1 and the exogenously delivered sesRNA plasmid in mCherry-positive cells isolated from the three groups. ** *p* < 0.01.

In the past several years, several ADAR-mediated RNA sensing methods have been developed including CellREADR [18], RADAR (RNA sensors using ADAR) [19], RADARS (reprogrammable ADAR sensors) [20], and ADAR-recruiting hairpin RNA sensors [21]. These technologies exploit the endogenous RNA-editing activity of ADAR to convert cell-type-specific RNA expression into detectable reporter signals. The principle involves designing sensor RNA (sesRNA) sequences that are partially complementary to the endogenous target RNAs. A stop codon “TAG” in frame is introduced near the center of the sesRNA sequence to create an A:C mismatch within the endogenous sequence “ACC”. This partial double-strand RNA (dsRNA) recruits the ADAR to convert “A” to “I” by deamination in the mismatched base pair (Figure 1a). “I” is recognized as “G” within the cell, allowing the subsequent translation of the effector gene following the sesRNA [22,23]. The ubiquitous presence of ADAR in cells eliminates the need to introduce exogenous components analogous to the CAS9 protein of the CRISPR-Cas9 system. Moreover, ADAR-based RNA sensing exhibits low cytotoxicity [18]. However, many aspects of CellREADR technology remain unclear, and its application in tracing stem cell differentiation has not been explored.

Spermatogonial stem cells (SSCs) hold great promise for applications in human reproduction and animal breeding [24,25]. To date, credible reports of stable SSC line establishment are limited to mouse, rat, and tree shrew [26,27,28]. Among these, mouse SSCs (mSSCs) are widely considered the most representative model, owing to their well-established culture systems and extensive biological characterization. Although we have previously achieved efficient knock-in of copGFP at the *Actb* locus in mSSCs [29], precise knock-in at most other loci of interest remains exceedingly challenging. First, mSSCs proliferate slowly and are refractory to conventional lipid-based transfection; even electroporation, while effective for certain targets, causes substantial cell loss given the limited cell numbers. Second, the homologous recombination (HR) pathway in mSSCs is relatively inefficient during the DNA damage response [30]. Third, for genes with restricted or differentiation-dependent expression, chromatin inaccessibility further compromises editing efficiency [31]. Given the simplicity and versatility of CellREADR, we evaluated its potential as a practical strategy for labeling and monitoring mSSCs.

We first investigated the effects of various factors on the efficiency of CellREADR, including the Woodchuck Hepatitis Virus Posttranscriptional Regulatory Element (WPRE), different mismatch types, and stop codon selection in sesRNA. Subsequently, we demonstrated the cell-type-specific discrimination capability of CellREADR in human embryonic kidney 293FT (HEK293FT) and HeLa cell lines. We further evaluated the accuracy on similar sequence detection. Finally, we implemented this technology in mouse embryonic stem cells (mESCs) and mSSCs to explore its potential in identifying cell types and monitoring differentiation.

## 2. Methods

### 2.1. sesRNA Design

All sesRNA sequences in this article were designed strictly following the steps outlined by Qian, Y et al. [18]. Briefly, the design criteria included: (1) choosing cell-type-specific RNAs, such as *Plzf* for mSSCs, to ensure that CellREADR activation is restricted to the target cell population; (2) targeting the coding regions (CDS) of the target genes for all sesRNAs used in this study—although targeting introns or untranslated regions (UTRs) is also permissible, and we chose CDS for consistency with the original design framework and to facilitate direct comparison across different targets; (3) designing sensor sequences of 200–300 nucleotides that contain appropriate CCA sites for efficient stop-codon conversion; and (4) selecting targets with sufficiently high expression levels in the target cells, as sensor efficacy is positively correlated with target RNA abundance. It should be noted that, based on our results, replacing the functional stop codon in sesRNA 5′-TAG-3′ with 5′-TGA-3′ is also feasible, if it could reduce the formation of complex secondary structures. The specific sequences are detailed in Supplementary Note S1.

### 2.2. Plasmid and Clone

All primers used in this study were synthesized by Tsingke Biotechnology Co. Ltd. (Beijing, China). The sesRNA plasmid backbones were derived from pCMV-PE2 (Addgene plasmid # 132775) and PLV-huGDNF (Addgene plasmid # 182946). All plasmid constructions followed standard molecular cloning procedures. The molecular reagents used included but were not limited to NheI-HF^®^ (NEB [ Ipswich, MA, USA], R3131), BamH1-HF^®^ (NEB, R3136), BsaI-HF^®^v2 (NEB, R3733), KpnI-HF^®^ (NEB, R3142), 2×TransStart^®^ FastPfu Fly PCR SuperMix(-dye) (TransGen Biotech [Beijing, China], AS231), Escherichia coli Trans1-T1 (TransGen Biotech, CD501), Zymoclean Gel DNA Recovery Kit (ZYMO RESEARCH [Irvine, CA, USA], D4002), EndoFree Plasmid Midi Kit (CWBIO [Beijing, China], CW2105S), ClonExpress Ultra One Step Cloning Kit (Vazyme [Nanjing, China], C115), and DNA Ligation Kit Ver.2.1 (Takara [Shiga, Japan], 6022).

### 2.3. Cell Culture

All cells were cultured at 37 °C with 5% CO_2_ and were mycoplasma-free. Different cell lines were maintained in diverse media compositions. Briefly, HeLa, HEK293FT, STO, STO-GDNF and MEF cells were cultured in DMEM (Gibco [Grand Island, NY, USA], 12100046) supplemented with 10% FBS (cytiva, sv30160) and 1% penicillin-streptomycin (Gibco, 15140122). Cells were digested with 0.25% trypsin-EDTA (Gibco, 25200) then passaged at a 1:3 ratio upon reaching confluence. MEF cells at passage 5 treated with 10 μg/mL mitomycin C (Sigma [Burlington, MA, USA)], M0503) for 2.5 h were feeder cells to support the growth of mESCs and mSSCs. mESCs were typically cultured on MEF feeder using N2B27 medium consisting of DMEM/F12 (Thermo Fisher Scientific [Waltham, MA, USA], 11330-032), neurobasal (Thermo Fisher Scientific, 21103-049), 1× N2 supplement (Thermo Fisher Scientific, 17502-048), 1× B27 supplement (Thermo Fisher Scientific, 12587-010), 2 mM L-glutamine (Gibco, 25030), 1× NEAA (Gibco, 15140), 100 μM 2-mercaptoethanol (Sigma, M7522), 1% penicillin-streptomycin (Gibco, 15140122), 10^3^ units/mL mLIF (Milllipore [Darmstadt, Germany], ESG1107), 3 μM CHIR 99021 (Tocris, 4423) and 1 μM PD 0325901 (Tocris [Bristol, UK], 4192). When transfection was required, mESCs originally cultured on MEF feeder need first to be pre-incubated after terminating digestion by DMEM supplemented with 10% FBS and 1% penicillin-streptomycin to remove feeder cells and then planted into poly-L-ornithine-laminin-coated wells. For preparing poly-L-ornithine-laminin-coated wells, briefly, the wells were coated with 0.01% poly-L-ornithine at 25 °C for more than 1 h, then continuously coated with 300 ng/mL laminin (Yeasen [Shanghai, China], 92602ES) again. Regarding mSSCs, we have described the exact establishment and culture method previously [29]. Briefly, mSSCs were established from 3-month-old C57BL/6 mouse through two-step digestion method: the first digestion system is 1 mg/mL Dnase1 (AppliChem [Darmstadt, Germany], A3778) plus 1 mg/mL collagenase1 (Gibco, 17100-017), and the second is 0.25% trypsin-EDTA. mSSCs were typically cultured on mitomycin C-treated MEF feeders with specific culture medium consisting of MEMα (Gibco, 12571), 2 mg/mL BSA (MP, 9078-46-8), 5 μg/mL insulin (Sigma, I2643), 30 nM Na_2_SeO_3_ (Sigma, 2114485), 25 μg/mL transferrin (Sigma, I2643), 10 μg/mL putrescine (Sigma, P5780), 2 mM L-glutamate (Gibco, 25030), 1 × lipoprotein (MP, 191476), 7.6 μeq/L free fatty acid cocktail, 5 ng/mL human bFGF (peprotech, 400-29), 20 ng/mL human GDNF (R&D, 212-GD), 20 μg/mL L-ascorbic acid (Sigma, A4544), 5.5 μM 2-mercaptoethanol (Sigma, M7522) and 1× Penicillin streptomycin. Fresh medium changes occurred every two days and cells were passaged at a 1:2 ratio every four days.

### 2.4. Plasmid Transfection

All transfection experiments were conducted in 24-well plates when the cell density was approximately 80%, and the fresh culture medium was replaced before transfection. Unless otherwise specified, the total amount of DNA transfected per well was 1 μg. HEK293FT cells were transfected with TransIntro^®^ PEI Transfection Reagent (GMP Grade) (TransGen Biotech, FT401). The transfection mixture consisted of 1 μg DNA + 3 μL PEI + 25 μL PEI buffer, incubated at room temperature for 15 min before transfection. For mESC transfections or co-transfections of HEK293 and HeLa cells, Lipofectamine™ 2000 (Thermo Fisher Scientific, 11668019) was used according to the manufacturer’s instructions. For STO and STO-G cell lines, Lipofectamine LTX (Invitrogen [Waltham, MA, USA], 15338100) were used according to the manufacturer’s protocol, and the optimized reagent composition is 2 μL LTX + 1 μg DNA +1 μL PLUS™ Reagent. Transfection efficiencies, determined by flow cytometry based on mCherry expression 48 h post-transfection, were >80% in HEK293FT cells and approximately 15% in STO cells under the optimized conditions. For mESCs, transfection was performed following the Lipofectamine 2000 manufacturer’s protocol without further quantification.

### 2.5. Flow Cytometry Analysis and Sorting

Forty-eight hours after transfection, cells were digested with 0.25% Trypsin-EDTA for 2 min at 37 °C, then terminated with DMEM + 10% FBS + 1% penicillin-streptomycin. The digested cell suspension was filtered through a 400-mesh filter membrane into specific flow cytometry tubes and transported and stored on ice until analysis. The raw data were analyzed using FlowJo V10 software. For sorting, the same procedure was followed, except that the collected cells were centrifuged at 600 g for 6 min, the supernatant was discarded, and the cells were reseeded into culture plates.

### 2.6. RNA Extraction and RT-qPCR

Total RNA was extracted from cells using TRIzol reagent (Thermo Fisher Scientific,15596026CN) following the manufacturer’s instructions. Then 1 μg of RNA underwent reverse transcription following the protocol (Vazyme, R323). The resulting cDNA was diluted fivefold and used as a template for qPCR. RT-qPCR analysis was performed using SYBR dye (Vazyme, Q712) in a LightCycler^®^ 96 Instrument. Gene-specific primers were designed by PrimerBank (harvard.edu) and are listed in the Supplementary Table S2.

### 2.7. Western Blot

Primary antibody against ADAR1 (Beyotime [Shanghai, China], AF6153, 1:1000) and β-actin antibody (Sigma, A5316, 1:2500) were used in this study. Cells from one well of a 12-well plate were collected and lysed using 190 μL 2× Laemmli Sample Buffer (Bio-Rad [Hercules, CA, USA], 1610737) supplement with 10 μL 355 mM 2-mercaptoethanol and 1× protease inhibitor cocktail (Mei5bio, MF182), followed by heating at 100 °C for 10 min. Then 5 μL lysates was loaded for SDS-PAGE (LabLEAD [Beijing, China], P41212) and run at 120 V for 70 min. The proteins were then transferred onto 0.45 μm PVDF membranes (Vazyme, E801-02-AA) using the Trans-Blot Turbo Transfer Starter System (Bio-Rad, 17001915). The PVDF membrane was blocked with 5% DIFCO^TM^ Skim Milk (BD, 232100) for 1 h at room temperature and then incubated with primary antibody overnight at 4 °C. Following three washes with TBST (Lablead, T7209), the membrane was incubated for 1 h at room temperature with secondary antibodies: HRP-labeled goat anti-rabbit IgG(H + L) (Biodragon [Beijing, China], BF03008, 1:2000) and HRP-labeled goat anti-mouse IgG(H + L) (Biodragon, BF03001, 1:2000). After incubation, the membrane was washed three more times with TBST. The result was imaged using BeyoECL Plus (Biyotime [Shanghai, China], P0018) with ChemiDOx XRS+ system.

### 2.8. Multiple Sequence Alignment (MSA) and Visualization

The cDNA and protein sequences of *mGdnf*, *huGDNF*, and *opti-cod huGDNF* were aligned by Clustal Omega (version 2.3.0)< EMBL-EBI. The visualization of the similarity matrix and sequence annotation derived from MSA was implemented by the pheatmap package in R and the Jalview (version 2.11.4.1) software.

### 2.9. Lentivirus Preparation

HEK293 cells were seeded in 10 cm dishes and grown until they reached approximately 80% confluence. The culture medium was replaced with antibiotic-free medium before transfection. The cells were transiently transfected with PEI using 8 μg of the sesRNA plasmid, 2 μg of pMD2.G (Addgene plasmid # 12259), and 4 μg of psPAX2 (Addgene plasmid # 12260). The medium was changed to fresh medium 16 h post-transfection. The supernatant was collected at 48 and 72 h post-transfection and centrifuged at 600 g for 6 min. The collected supernatant was filtered through a 0.45 μm filter and transferred to specific lentivirus centrifuge tubes. An adequate volume of PEG8000 (Beyotime, ST483) was incorporated to reach a final concentration of 8.5% (*m*/*v*). The mixture was then thoroughly combined at 4 °C for 4 h, followed by centrifugation at 10,000 rpm at 4 °C for 20 min. The supernatant was discarded, and the pellet was resuspended in one-tenth of the volume of the collected supernatant using MEMα. The lentivirus was aliquoted into 50 μL volumes and stored at −80 °C.

### 2.10. mSSC Lentivirus Infection

The digested mSSCs suspension was seeded into new wells at a 1:2 ratio based on the original culture plate area and pre-incubated at 37 °C with 5% CO_2_ for 15 min to remove some of the MEF feeders. The pre-incubated cell suspension was collected, centrifuged, and the supernatant was discarded. The cells were then seeded at 500,000 cells per well in laminin-coated 12-well plates. After 1 day of culture, the lentivirus suspension mixed with polybrene (1:1000) (Yeasen, 40804ES) was added to the mSSC culture medium. The medium was replaced 1 day after infection. The transduction efficiency, determined by mCherry expression via flow cytometry, was approximately 80% after two rounds of infection (Supplementary Figure S6a,b).

### 2.11. RA Treatment of mSSCs

A total of 20,000 feeder-free mSSCs was seeded into a laminin-coated 24-well dish and cultured for 24 h. The medium was then replaced with fresh medium containing final concentration of 100 nM RA (Sigma, 302-79-4). After treatment for 24 h, the fresh medium without RA was replaced. Notably, RA is photosensitive and easy to degrade; it was prepared immediately before use and protected from light throughout the experiment.

### 2.12. Cell Plating and Immunofluorescence Staining

For immunofluorescence analysis, sterile coverslips were placed into 24-well plates and pre-coated with laminin. Cells were then seeded at a density of 2 × 10^4^ cells per well and incubated for 24 h. Subsequently, the cells were fixed by adding 200 μL of 4% (*m*/*v*) paraformaldehyde (Thermo Fisher Scientific, 169650010) to each well and incubating for 15 min at room temperature. After fixation, the cells were washed three times with DPBS (Gibco, 21600010) for 5 min per wash. The cells were then permeabilized with 200 μL of 0.1% (*m*/*v*) Triton X-100 (Sigma, 234729) per well for 15–20 min at RT, followed by three washes with DPBS. The cells were blocked with 200 μL of 5% BSA for 1 h at room temperature. Primary antibody against PLZF (R&D, AF2944, 1:200) was used in this study. Then 50 μL of diluted primary antibody solution was added to each well and incubated overnight at 4 °C. After washing three times with DPBS, the cells were incubated with 200 μL of donkey anti-goat IgG (H + L) cross-adsorbed secondary antibody, Alexa Fluor™ 488 (Thermo Fisher Scientific, A11055, 1:1000) per well for 1 h at room temperature in the dark. The cells were then incubated with DAPI (Beyotime, C1002, 1:1000) for 10 min. After completion, coverslips were carefully transferred onto glass slides dripped with antifade mounting medium (Beyotime, P0126).

### 2.13. Statistical Analysis

All data were obtained from three independent biological replicates. Data analysis was performed using GraphPad Prism 8. Data are presented as mean ± SD. Independent sample two-tailed t-tests or one-way analysis of variance (ANOVA) were conducted to assess significance. Statistical significance was defined as * *p* < 0.05, ** *p* < 0.01, *** *p* < 0.001, and ns (not significant) for *p* > 0.05.

## 3. Results

### 3.1. Validation and Further Characterization of CellREADR

Based on the design principles outlined in a previous study, we constructed the reporter system with mCherry as the indicator and copGFP as the effector (Figure 1b), with sesRNA targeting the endogenous *EEF1A1* (ses-*EEF1A1*) as the primary test case [18]. We replaced the sesRNA sequence with a short linker containing or lacking the stop codon in frame as the negative (ses-negative) and positive control (ses-positive), respectively. We noted that the WPRE, which is usually used in viral vectors to enhance gene expression by post-transcriptional regulation [32], is present in the CellREADR plasmid construct [18]. Its absence in RADAR, RADARS, and ADAR-recruiting hairpin RNA sensor technologies may stem from the typical use of WPRE to enhance virus-related infectivity [33,34], while its effect on the performance of ADAR-mediated RNA sensing remains undiscussed. To explore this, we further incorporated the WPRE into both the negative control (ses-negative-W) and the ses-*EEF1A1* system (ses-*EEF1A1*-W).

The ses-negative construct was expected to yield a low percentage of green cells within the red population upon delivery into HEK293FT cells, likely due to the leaky expression of cop-GFP. This negative construct generated a background conversion rate of at most 1%. Accordingly, the conversion rates achieved for the ses-negative-W, ses-EEF1A1, ses-EEF1A1-W, and ses-positive constructs were 3.99 ± 0.50%, 18.50 ± 0.95%, 26.23 ± 3.15%, and almost 100%, respectively (Figure 1c,d and Supplementary Figure S1). These results not only validated the effect of CellREADR but also demonstrated that WPRE integration significantly enhances the positive signal generated by CellREADR, despite that it also slightly increases the background signal. Based on this, we keep WPRE in the plasmid for subsequent experiments.

Additionally, we observed that mCherry fluorescence intensity was markedly decreased in ses-EEF1A1 and ses-EEF1A1-W cells compared with that in ses-positive cells, whereas no obvious change was observed in ses-negative or ses-negative-W cells (Figure 1e). We reasoned that the RNA sensing process likely triggered RNA degradation, supported by the observation that ADAR1 participates in RNAi by forming a complex with Dicer [35]. To investigate this further, we transfected the ses-negative-W, ses-*EEF1A1*-W, and ses-positive plasmids into HEK293FT cells and sorted cells with strong mCherry signal. RT-qPCR with validated copGFP-targeted primers revealed that the plasmid-derived exogenous sequence in the ses-EEF1A1-W group was reduced by 2.6- to 4.0-fold relative to the ses-negative-W and ses-positive groups. However, no significant differences in endogenous EEF1A1 expression were observed among these groups (Figure 1f and Supplementary Figure S2). These data suggest that RNA sensing preferentially destabilizes the exogenous sesRNA-containing transcript rather than the endogenous target.

### 3.2. Mismatch Optimization and Mutation Detection of CellREADR

**Figure 2:**
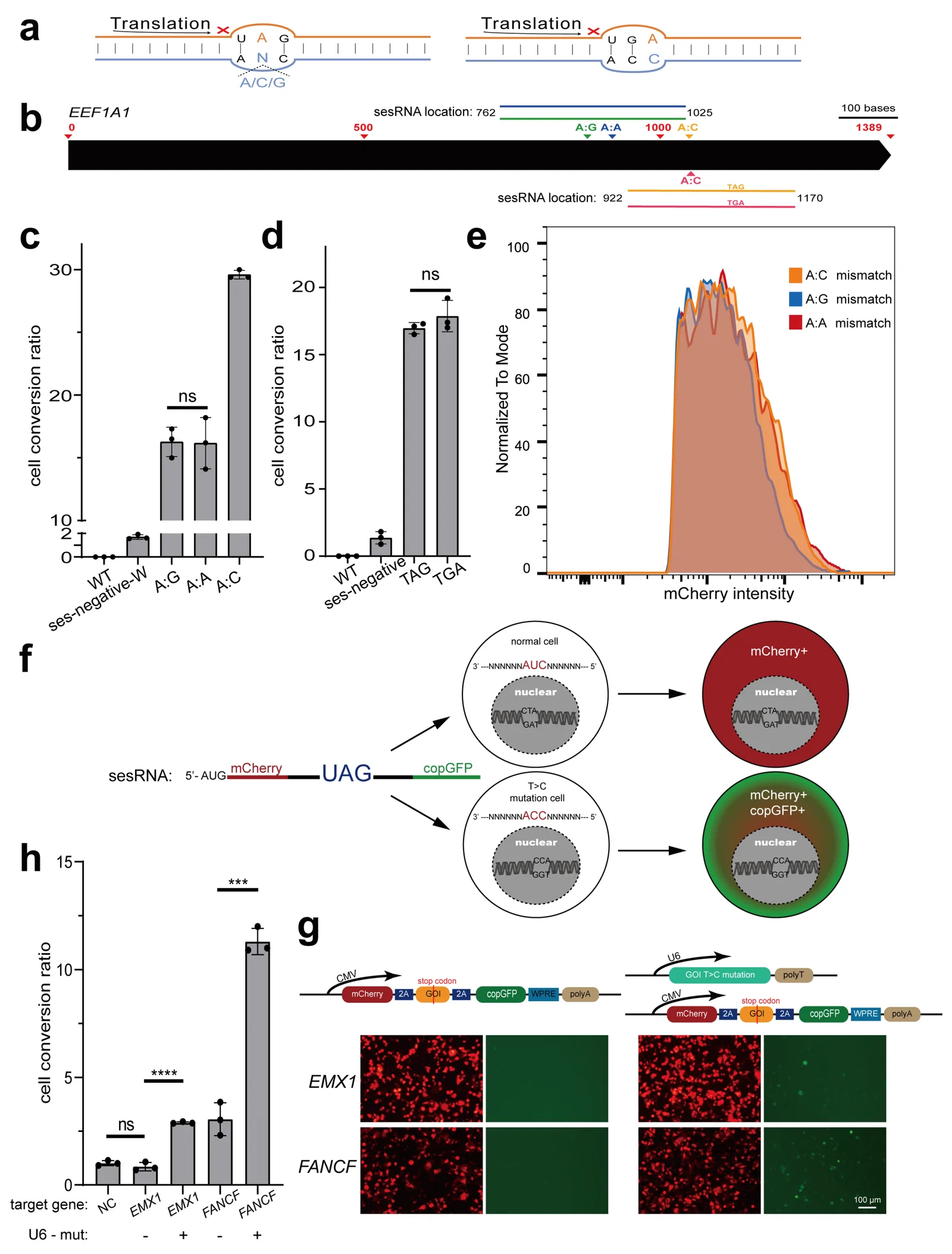
The optimal mismatch designs of CellREADR and its ability on gene mutation detection. (**a**) Schematic diagram of A:N mismatches and the design of the stop codon UGA. (**b**) According to the CDS region of *EEF1A1*, the designed sesRNA of A:A, A:G, A:C mismatches and UGA stop codon correspond to the positions on the sequence. (**c**,**d**) A: N mismatch and different stop codons statistics according to the results of flow cytometry. (**e**) A comparison of the fluorescence intensities of mCherry among A:A, A:G, A:C mismatch groups. (**f**) Schematic diagram of detecting genetic mutation in cells using CellREADR. (**g**) *EMX1* and *FANCF* genes were selected for mutation detection simulation. The left figures indicate cells transfected only with the sesRNA vector, while the right figures indicate cells co-transfected with both the sesRNA and gene mutation simulation vectors. GOI denotes the gene of interest, which refers to *EMX1* and *FANCF* in this context; scale bar is 100 μm. (**h**) Cell conversion ratio was counted from flow cytometry analysis in (**g**). *** *p* < 0.001, **** *p* < 0.0001. The red vertical lines indicate that a stop codon has been artificially introduced to create a mismatch.

ADAR-mediated adenosine deamination preferentially occurs in the A-C mismatch site [36,37], but alternative mismatches must be considered when the endogenous RNA sequence lacks suitable “CCA” site to design sesRNA (Figure 2a). To investigate this, we designed a single sesRNA variant harboring both A:A and A:G mismatches. This design ensures that the two mismatch types share an identical sequence context and secondary structure, thus eliminating confounding effects arising from differences in nucleotide abundance or variations in RNA secondary structure (Figure 2b).

The cell conversion rates of A:A and A:G mismatches were 16.50 ± 0.95% and 15.80 ± 1.75%, respectively, significantly lower than that of the A:C mismatch, which was 29.60 ± 0.35% (Figure 2c). There was no significant difference between A:A and A:G mismatches. This shows that A:C mismatches are optimal, but A:A and A:G mismatches are viable alternatives when A:C mismatches are not feasible. Moreover, sesRNA of the A:A and A:G mismatches also resulted in the attenuation of mCherry fluorescence intensity like the sesRNA of the A:C mismatch (Figure 2e and Supplementary Figure S3a).

Additionally, we note that both the “TAG” and “TGA” stop codons can form A:C mismatches with “CCA” (Figure 2a). To test whether these two stop codons can be used in sesRNA and whether their uses result in any difference, we modified the ses-*EEF1A1* sequence by replacing “TAG” of sesRNA with “TGA” (Figure 2b) and found no difference in the cell conversion ratio (Figure 2d). This suggests that the “TGA” stop codon can be tried when the use of “TAG” is not satisfying.

Previous studies have demonstrated that RADAR can detect mutations at specific sites despite low sensitivity [19]. To explore the ability of CellREADR in mutation detection (Figure 2f), we selected two housekeeping genes in HEK293FT, *FANCF* and *EMX1*, which commonly serve as the targets in prime editing [38,39]. We first generated plasmid constructs from which mRNA fragments of the gene of interest (GOI) containing a single-base mutation can be transcribed under the U6 promoter. Constructs of sesRNA targeting the mutant region of GOI mRNAs, ses-*FANCF*-W and ses-*EMX1*-W were accordingly designed and generated. HEK293FT cells were transfected with the ses-RNA plasmid with or without the plasmid for the mutant GOI (Figure 2g). In the absence of the mutant plasmids, the ses-*EMX1*-W and ses-*FANCF*-W groups exhibited cell conversion ratios of 0.85 ± 0.19% and 2.90 ± 0.04%, respectively. In the presence of the mutant plasmids, the positive rates significantly increased to 3.07 ± 0.77% and 11.30 ± 0.61% for ses-*EMX1*-W and ses-*FANCF*-W, respectively (Figure 2h and Supplementary Figure S3b). These results indicate that mutant mRNAs can be detected by CellREADR although the sensitivity warrants further investigation.

### 3.3. Distinguishing Two General Cell Lines by CellREADR

**Figure 3:**
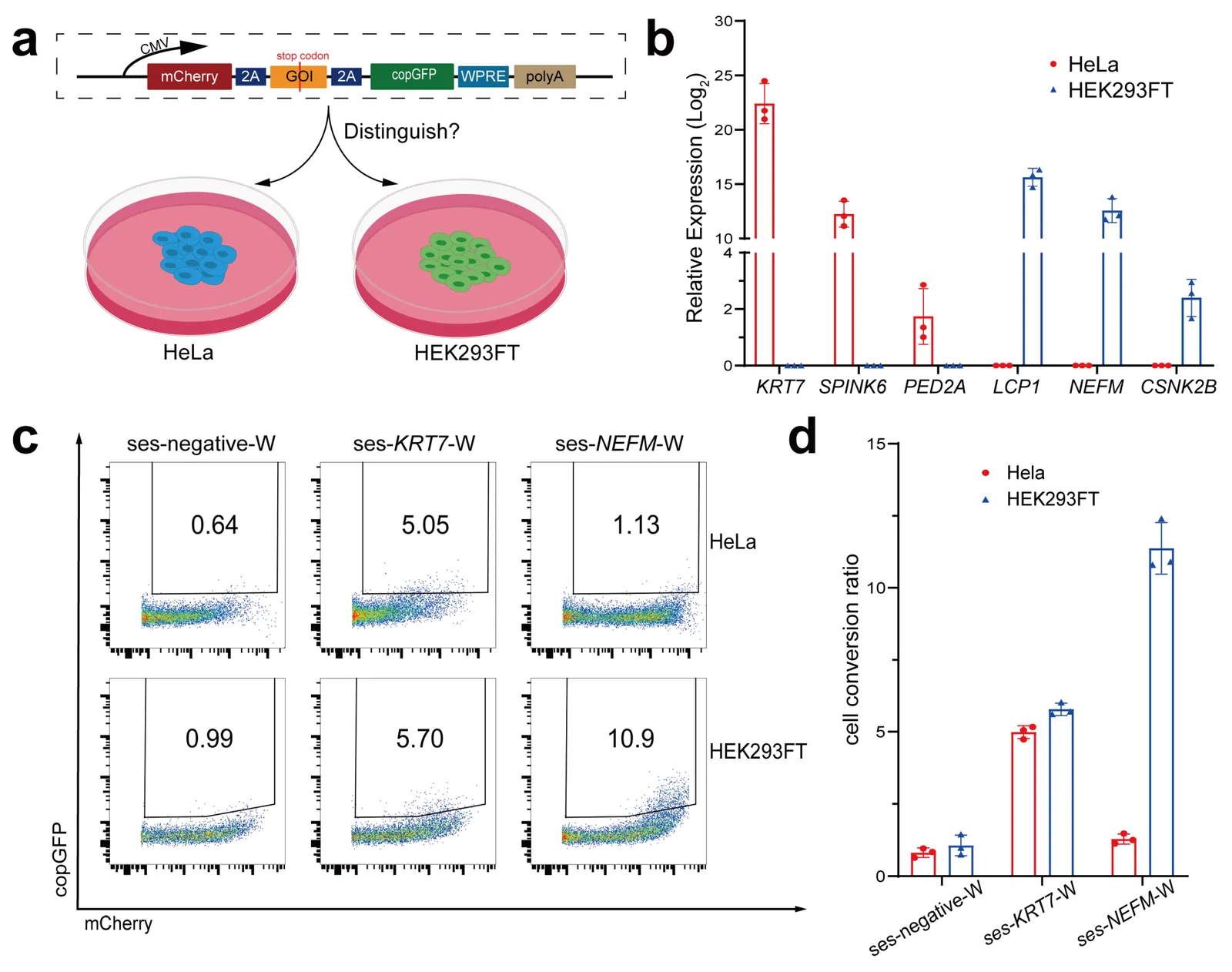
Distinguishing two general cell lines by CellREADR. (**a**) Schematic diagram of the design of distinguishing HEK293FT and HeLa cells using CellREADR. (**b**) Relative expression levels of *NEFM*, *LCP1*, *CSNK2B*, *SPINK6*, *KRT7*, and *PDE2A* genes in HEK293FT and HeLa cells. (**c**) The flow analysis result of assessing the cell conversion ratio of ses-*KRT7*-W and ses-*NEFM*-W in HEK293FT and HeLa cells. (**d**) Statistical results of the cell conversion ratio of ses-*KRT7*-W and ses-*NEFM*-W in HEK293FT and HeLa cells based on (**c**), *n* = 3. The red vertical lines indicate that a stop codon has been artificially introduced to create a mismatch.

The sesRNA designed targeting *Fezf2* (zinc-finger transcription factor) and *Ctip2* (B cell leukemia/lymphoma 11B) achieved >90% specificity in distinguishing two types of neurons in mouse cortex [18]. We assessed the performance of CellREADR in distinguishing HeLa and HEK293FT cell lines to further assess its suitability for detecting cell identity in common cell lines (Figure 3a). By comparing the transcriptomic profiles of these two cell lines, we selected six candidate genes that display high specificity in their expression patterns: *KRT7*, *SPINK6*, and *PED2A* exhibiting HeLa-specific expression, while *LCP1*, *NEFM*, and *CSNK2B* show preferential expression in HEK293 cells (Supplementary Table S1) [40]. Based on both expression specificity and sesRNA design feasibility, *KRT7* was selected as the HeLa-specific biomarker, whereas *NEFM* was chosen as the HEK293FT-specific biomarker (Figure 3b).

We subsequently designed ses-*KRT7*-W and ses-*NEFM*-W, constructed the corresponding expression vectors, and delivered the plasmids into both Hela and HEK293FT cells. While ses-*KRT7*-W showed comparable positivity rates in both cell types, ses-*NEFM*-W exhibited clear cell-type specificity. Specifically, ses-*NEFM*-W demonstrated only background-level signals (similar to the ses-negative-W group) in HeLa cells, but achieved an average 12% positivity rate in HEK293FT cells (Figure 3c,d). These results indicate that CellREADR can be used to discriminate different cell lines given that marker genes are reasonably selected and the performances of corresponding sesRNAs are experimentally validated.

### 3.4. Limited Accuracy of CellREADR for Targeting Similar Sequences

**Figure 4:**
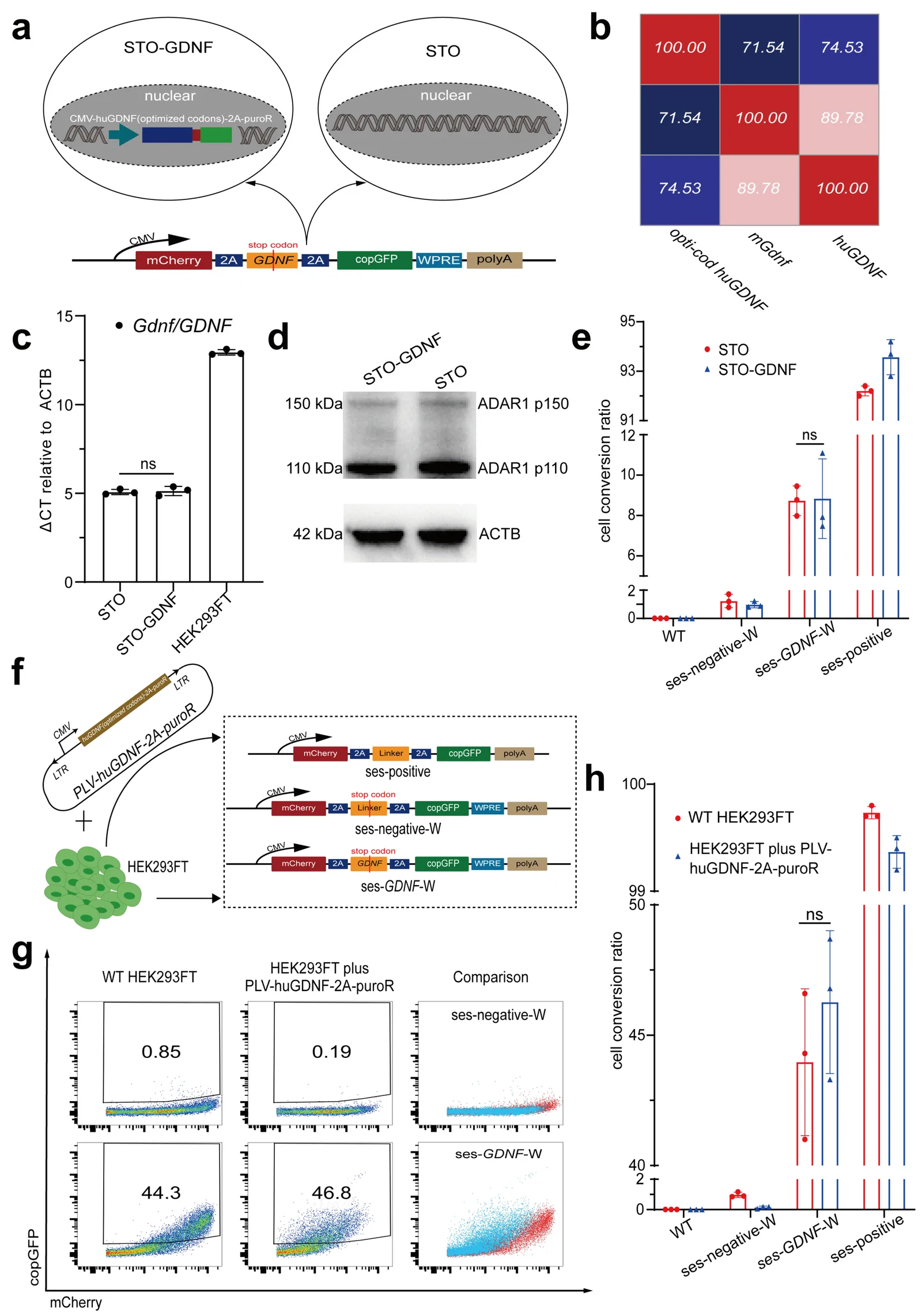
Measuring the accuracy of similar sequences detection by CellREADR. (**a**) Schematic diagram illustrating the distinction between STO and STO-GDNF cells. (**b**) DNA sequence similarity matrix of *mGdnf*, *huGDNF* and *opti-cod huGDNF*. (**c**) Relative expression levels of endogenous Gdnf/GDNF in STO, STO-GDNF, and HEK293FT cells, with Actb/ACTB as a reference. (**d**) Protein expression level of ADAR1 detected in the indicated cells, with ACTB as a reference. (**e**) Statistics of cell conversion rate after ses-*GDNF*-W transfection of STO and STO-GDNF cells, *n* = 3. (**f**) Schematic diagram of the design in HEK293FT. Here, in one group, the PLV-huGDNF-2A-puroR plasmid was transiently transfected into cells along with other sesRNA-related plasmids which include ses-positive, ses-negative-W and ses-*GDNF*-W. (**g**) Flow cytometry results for ses-negative-W and ses-*GDNF*-W groups, illustrating the cell conversion ratio and comparing fluorescence intensity within the same group across the two cell populations. (**h**) Statistical results of the cell conversion ratio, *n* = 3. The red vertical lines indicate that a stop codon has been artificially introduced to create a mismatch.

It is a common practice in molecular biology to express and detect homologous and/or codon-optimized transgenes in cells, and also to readily distinguish expressing cells from the bulk population. We wondered whether CellREADR can be used for this application. We first derived a STO cell line that expresses a codon-optimized human *GDNF* (*opti-cod huGDNF*) stably, here referred to as STO-GDNF (Figure 4a and Supplementary Figure S4a,b). Based on the *opti-cod huGDNF* sequence, we designed the corresponding sesRNA (ses-*GDNF*-W). By aligning the sequences of mouse *Gdnf* (*mGdnf*), human *GDNF* (*huGDNF*), and *opti-cod huGDNF*, we found that the sequence similarities between *mGdnf* and *opti-cod huGDNF*, as well as between *huGDNF* and *opti-cod huGDNF*, were 71.54% and 74.53%, respectively (Figure 4b). Alignment of ses-*GDNF*-W with endogenous human and mouse mRNA sequences identified many mismatched sites within the coding regions. The mismatch formed between *huGDNF* and ses-*GDNF*-W was A:A (Supplementary Figure S4c), and RT-qPCR confirmed abundant expression of endogenous levels of endogenous *Gdnf*/*GDNF* in STO, STO-GDNF, and HEK293FT cells (Figure 4c). Moreover, both STO and STO-GDNF cells exhibited abundant and comparable ADAR1 protein expression (Figure 4d), indicating that differences in CellREADR activity would be unlikely to arise from variations in ADAR availability.

To determine whether CellREADR could selectively detect the codon-optimized transcript, ses-*GDNF*-W was introduced into both STO and STO-GDNF cells using the optimized transfection system (Supplementary Figure S5a,b). Surprisingly, both cell lines exhibited nearly identical positive rates, 8.73 ± 0.74% in STO and 8.83 ± 1.97% in STO-GDNF (Figure 4e and Supplementary Figure S5c). We concluded that multiple mismatches between sesRNA and the endogenous target sequence do not prevent dsRNA formation or impair the efficiency of the RNA sensing system.

To determine whether this lack of discrimination was specific to STO cells, we sought to validate the finding in an independent cellular context so we conducted experiments in HEK293FT cells. As no HEK293FT-GDNF cell line analogous to STO-GDNF was available, we transiently expressed opti-cod huGDNF in these cells (Figure 4f). Results showed no significant difference in cell conversion rate between HEK293FT cells transiently expressing opti-cod huGDNF and wild-type (WT) HEK293FT cells. The positive rates for these two groups were 46.27 ± 2.74% and 44.00 ± 2.82%, respectively (Figure 4g,h and Supplementary Figure S5d). These results reflect the tolerance of the system to extensive sequence mismatches independent of cell types.

### 3.5. Stem Cell Lines Identification by CellREADR

**Figure 5:**
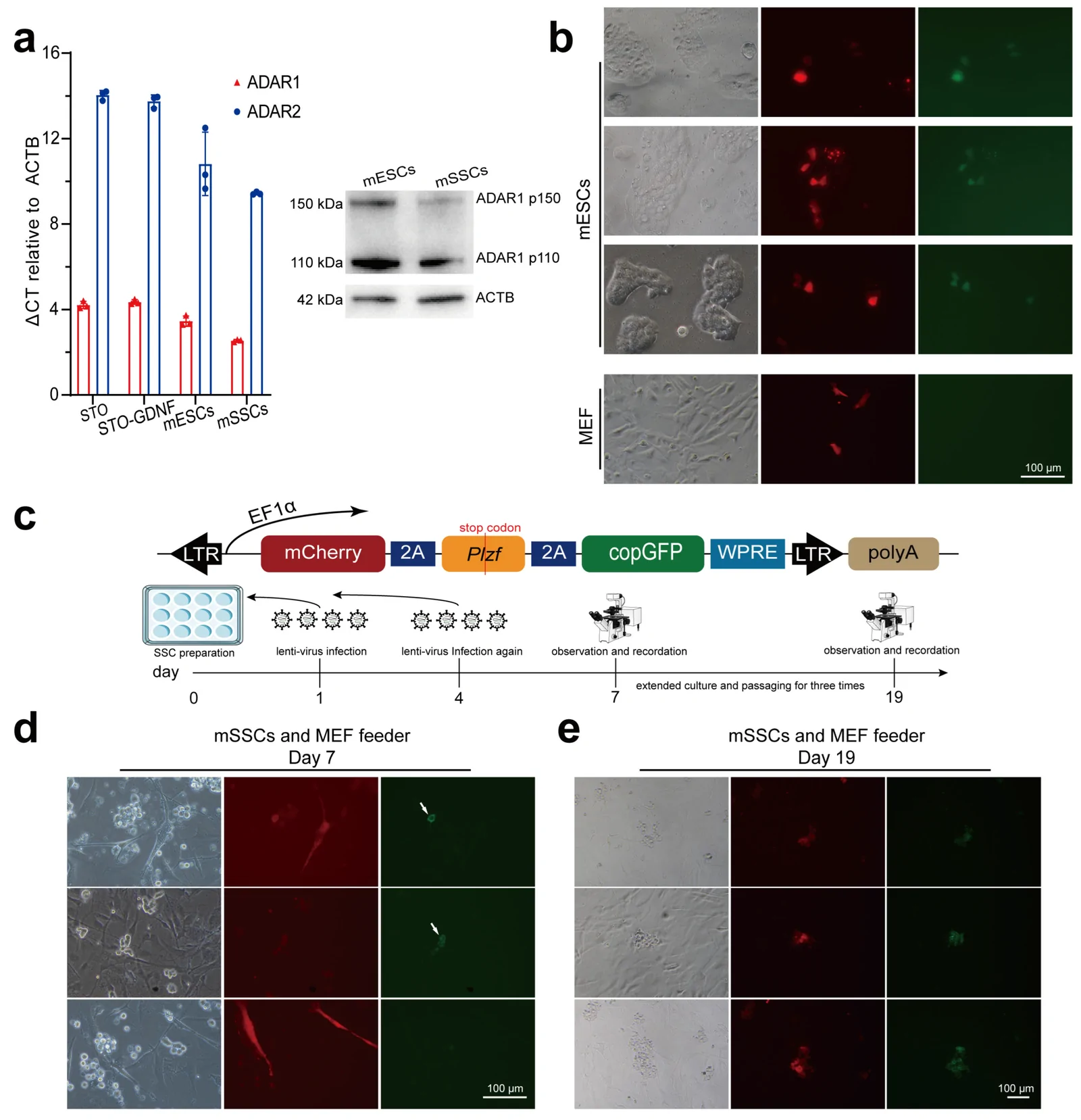
Exploring the feasibility of CellREADR in stem cell lines. (**a**) Relative mRNA expression levels of endogenous Adar1 and Adar2 in mESCs and mSSCs compared with those in STO and STO-GDNF cells, and ADAR1 protein expression levels in mESCs and mSSCs, with ACTB used as a reference. (**b**) The ses-*Klf4*-W construct was transfected into cells to specifically mark mESCs, with MEF cells used as the negative control. The white arrows point to the location of the mESCs that are specifically labeled, whereas in MEF cells, there is no effector signal at a scale of 100 μm. (**c**) The sesRNA targeting *Plzf* was designed to prepare lentivirus for mSSCs infection. The day of cell preparation of mSSCs was recorded as day 0, followed by lentiviral infection on day 1 and day 4, and recorded and observed on day 7 and day 19, respectively. (**d**) Fluorescence images on day 7; white arrows indicate the locations of mSSCs that are specifically labeled by CellREADR. And the MEF cell, serving as the feeder layer and negative control at the bottom of the well and exhibiting a fibrous morphology, shows no cell conversion signal; scale bar is 100 μm. (**e**) Fluorescence images on day 19 of the labeled mSSCs. Those cells formed clusters during extended culture while maintaining fluorescence labeling; scale bar is 100 μm. The red vertical lines indicate that a stop codon has been artificially introduced to create a mismatch.

To assess the feasibility of CellREADR in stem cell identification, we first tested its performance in mESCs with mouse embryonic fibroblast (MEF) cells as a negative control. We first examined the mRNA and protein expression of ADAR1 and ADAR2 in several cell lines including mESCs and mSSCs (Figure 5a). Both ADAR1 and ADAR2 exhibited high expression in mESCs, indicating that mESCs possess the molecular machinery necessary for effective CellREADR application. Then the classic marker genes *Oct4* and *Klf4* of mESCs were chosen as targets [41], and the corresponding sesRNAs, ses-*Oct4*-W and ses-*Klf4*-W, were designed. Two days after transfection of sesRNA plasmids into mESCs and MEF cells separately, we observed the cell conversion signal exclusively present in mESCs using ses-*Klf4*-W (Figure 5b), but ses-*Oct4*-W was invalid in both cell types.

Subsequently, we tested the feasibility of CellREADR in mSSCs, in which the two isoforms of ADAR1 are also detected as in mESCs (Figure 5a). Transcription factor PLZF is a specific marker for mouse undifferentiated spermatogonia, including mSSCs, and is not expressed in somatic cells in the testis or in MEF cells that are used as feeder cells in mSSC culture [42], and we designed ses-*Plzf*-W according to its mRNA. Because SSCs cannot be efficiently transfected with conventional lipid-based reagents, we used lentivirus to deliver ses-*Plzf*-W into mSSCs [43]. Moreover, the CMV promoter has low activity in mSSCs, so we replaced it with the EF1α promoter (Figure 5c).

mSSCs are routinely maintained on MEF feeder cells. At the beginning of the experiment, mSSCs were plated on a laminin-coated dish with a small number of MEF cells co-cultured as the negative control. Three days after twice lentiviral infections, copGFP expression was observed in a small number of mSSCs but not in any of the MEF cells (Figure 5c,d), which means mSSCs were specifically labeled by CellREADR. Given that the expression cassettes in lentiviral vector can be integrated into the host genome [44], durable long-term expression of the exogenous gene is achieved. We next assessed whether the copGFP-positive mSSCs could be maintained during long-term culture. After three passages, small clusters of mSSCs remained positive for both mCherry and copGFP (Figure 5e), and quantitative analysis revealed that these cells accounted for approximately 25% of the total population (Supplementary Figure S6a,b). Notably, this stable expression was maintained for up to two months (Supplementary Figure S6c), confirming long-term labeling stability. Collectively, these results indicate that mSSCs can be specifically labeled by CellREADR with long-term stability.

### 3.6. Monitoring mSSCs Differentiation by CellREADR

**Figure 6:**
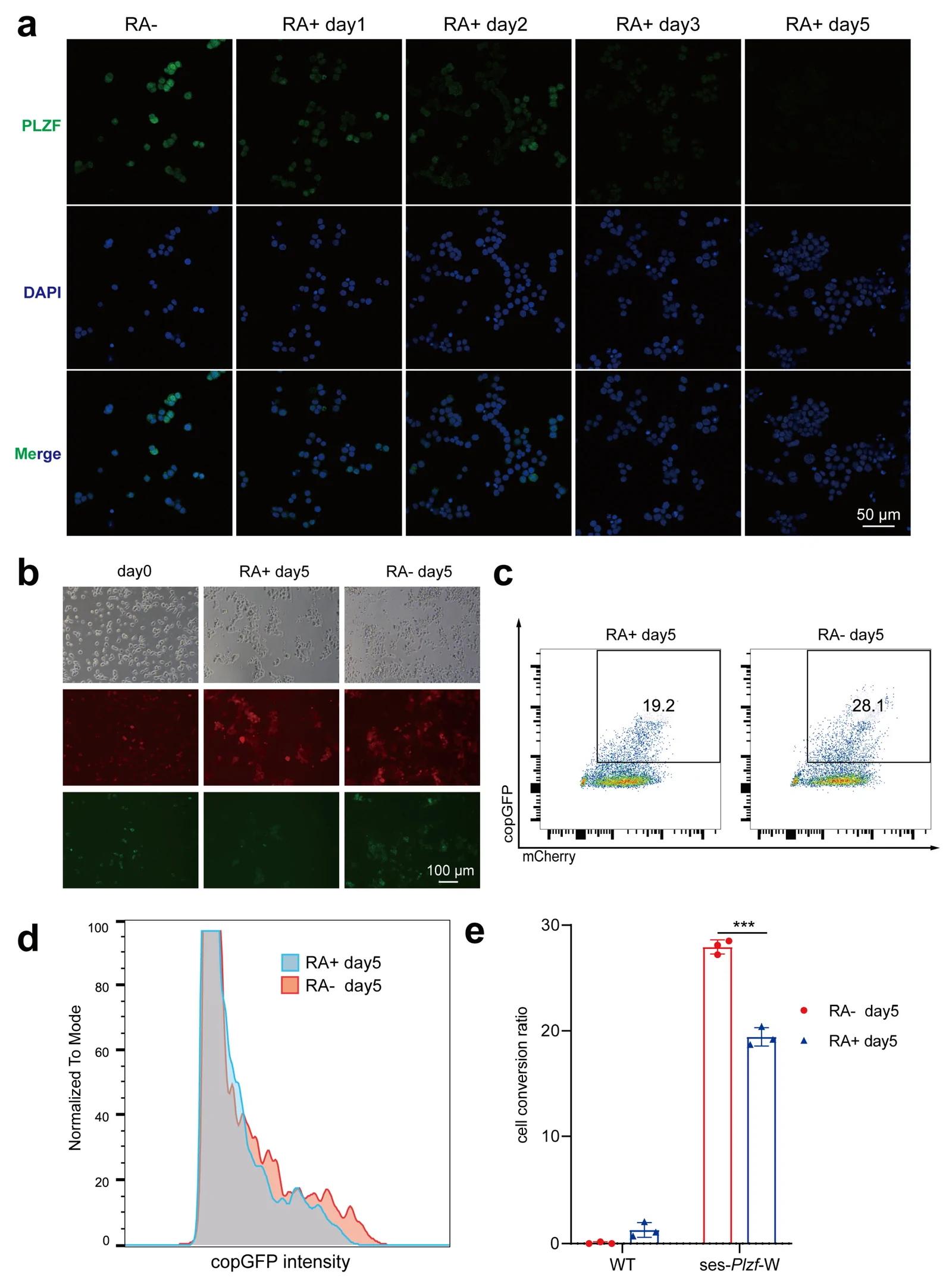
Monitoring mSSCs states transition by CellREADR. (**a**) Assessing the protein levels of PLZF in mSSCs before and after RA induction for day 1, 2, 3 and 5 using immunofluorescence staining; scale bar is 50 μm. (**b**) Fluorescence images of CellREADR-labeled mSSCs (Figure 5) on day 0, and on day 5 with or without RA treatment. After 5 days of RA treatment, mSSCs formed larger colonies and became more flattened; scale bar is 100 μm. (**c**) Flow cytometry results for RA-treated and untreated groups. (**d**) Comparison of copGFP fluorescence intensity between RA treatment group and non-treatment group. (**e**) Statistical results of the cell conversion ratio in RA-treated and untreated groups. *** *p* < 0.001.

Our previous study showed that the cultured mSSCs could be induced by RA to differentiate into spermatocytes, with *Plzf* RNA expression gradually reduced [45]. Consistent with this, PLZF protein expression diminished after 5 days of induction (Figure 6a). To assess the sensitivity of CellREADR in monitoring cellular differentiation, we plated labeled mSSCs (Figure 5e) onto a laminin-coated plate. These cells were treated with RA to induce differentiation, with untreated cells as a control. WT mSSCs served as a parallel control.

Five days after inducing, fluorescence microscopy and flow cytometry analysis revealed a significant decrease in both the percentage of copGFP-positive cells and the mean fluorescence intensity in the RA-treated mSSCs compared to controls (Figure 6b–d). Moreover, the cell conversion rate in the RA-treated group was 19.43 ± 0.87%, markedly lower than the 27.96 ± 0.67% rate observed in the untreated group (Figure 6e). The persistence of residual copGFP signal in cells 5 days after RA treatment when PLZF is undetectable is probably due to the asynchronous degradation process and long life-span of copGFP. Collectively, these results indicate that CellREADR enables monitoring of mSSCs differentiation status, even in the presence of delayed effector signal clearance.

## 4. Discussion

Cellular identity characterization and dynamic monitoring, also known as lineage tracing, is a common technology in biomedical research. We are particularly interested in how to identify mammalian SSCs and monitor their self-renewal and differentiation in vitro. Although mSSCs can be readily expanded and induced to differentiation into early spermatocytes [45,46], SSCs from large animals such as humans and pigs are extremely hard to culture due to slow growth and low abundance among all testicular cells [47,48]. Therefore, it is urgent for us to develop live cell tracking techniques that are simple and reliable. To this end, we test the applicability of the CellREADR system that has been reported recently [18]. We first tested its performance in commonly used cell lines, HEK293FT and Hela cells, and then in stem cells such as mESCs and mSSCs. Our results confirmed the potential value of this technology for future applications while also revealing several limitations that must be overcome before it can become more practical.

We investigated the effects of other features of CellREADR such as the use of A:A and A:G mismatches instead of A:C, and the use of stop codon TGA instead of TAG. Our results show that CellREADR performs well under these conditions; therefore, we provided more choices to satisfy the basic requirement of the system. Moreover, we confirmed that CellREADR has potential as an effective approach for genetic diagnosis through specific detection of mutations at defined loci. Although CellREADR cannot distinguish between highly homologous sequences, our data show that an efficient sesRNA can be used to detect homologous sequences from different species or designed artificially as long as the homology between the sesRNA and a target RNA are high enough; the threshold may need to be determined in a case-dependent way.

One concern is that the effector copGFP in the ses-RNA construct can be precociously expressed due to non-specific targeting of the ses-RNA by other partially complementary RNAs or by alternative translation initiation. In this study, we repeatedly found that the ratios of copGFP+ cells among mCherry+ cells (conversion rates) are satisfyingly low in the absence of target RNAs even if the ATG start codon is included at the beginning of the copGFP coding sequence (Figure 1b). This indicates that the method is highly specific. However, it is not sufficiently sensitive, as the conversion rates remained below 50% for all examined cases. The low sensitivity of CellREADR may result from the low expression of ses-RNA. We found that incorporating WPRE in sesRNAs can enhance their stability and/or translation efficiency. Indeed, the conversion rate increased from 18% to 26% (Figure 1d). One possible cause for the low-level expression of sesRNAs is RNAi-induced RNA degradation as double stand RNA structure is inevitable. We not only confirmed the existence of RNAi between sesRNAs and target RNAs but found that the expression level of sesRNAs but not that of the endogenous target RNAs was significantly reduced as indicated by the reduced mCherry intensity and reduced transcript abundance of copGFP (Figure 1e,f). Other explanations for the low sensitivity include the heterogeneous expression of the cellular RNAs due to cell cycle progression or other random events inherent to the system that warrants further investigations. From a technical implementation standpoint, CellREADR does not require genomic modification, exogenous protein expression, or complex delivery strategies beyond standard plasmid transfection, making it relatively straightforward to establish in vitro.

In the present study, we first showed that HEK293FT and HeLa cells can be identified by using CellREADR if target genes are carefully selected based on their differential expression and experimentally tested. However, its performance in stem cells has not been evaluated. We then reported that mESCs and mSSCs can be identified based on the expression of certain marker genes. In particular, mSSCs can be identified among co-cultured MEF cells and their RA-induced differentiation can be observed based on the reduction in the conversion rate. We acknowledge that a systematic quantitative assessment of CellREADR labeling specificity—such as flow cytometric co-staining of copGFP with established SSC markers (e.g., PLZF or GFRA1)—was not performed in the present study. Additionally, we did not systematically evaluate the impact of CellREADR on SSC-specific functions, including proliferation kinetics, self-renewal efficiency, or apoptosis rates. While our functional observations—including long-term clonal propagation and RA-induced differentiation—together with previous transcriptomic evidence [18] suggest minimal biological perturbation and support selective labeling of mSSCs, future studies incorporating quantitative flow cytometric validation and functional assays will be valuable to further confirm the system’s specificity and neutrality. Our results suggested that CellREADR should not be intended as a universal method for detecting gene expression as it often failed on a particular target gene that seemed promising before experimental test. For example, Oct4, the well-known stem cell marker gene, failed in mESC identification. However, CellREADR can be used as a valuable tool for cell type/state identification as long as the right target gene is selected.

Another concern for CellREADR is that using RNA-level rather than protein-level differences as the basis for cell identity annotation can be inappropriate under certain specific circumstances. Protein expression depends on RNA templates transcribed from DNA, meaning that the RNA of a cell marker must appear prior to the protein. Yet in some physiological events, RNA translation significantly lags behind DNA transcription—a delay caused by dedicated translational repression mechanisms rather than simply reflecting the sequential order of transcription and translation. Hence, the presence of RNA cannot be equated with the presence of protein. For instance, genes required for late spermatid development are transcribed in advance and stored in a translationally repressed state as messenger ribonucleoproteins (mRNPs) [49]. This specialized phenomenon is typically considered only in studies of specific cell types and particular developmental processes. In general, under steady-state conditions, cellular RNA and protein levels largely correspond, making it acceptable to infer protein levels from mRNA abundance. Thus, specific detection and recognition of marker RNAs represent another reasonable strategy. It should be noted that “steady state” is broadly defined here: during cell proliferation, differentiation, or other biological events, as long as intracellular RNA or protein levels remain relatively stable over an extended period, the cell can be considered to be at steady state [50].

It is important to distinguish between the proof-of-principle validation of CellREADR and its immediate applicability to in vivo lineage tracing. The present study provides a proof of concept that CellREADR can specifically label target cells in vitro and in cultured mSSCs based on endogenous RNA signatures, suggesting considerable specificity with relatively limited off-target effects under the tested conditions. Notably, viral delivery of CellREADR has been shown to confer specific cell-type access in mouse and rat brains and in ex vivo human brain tissues, and CellREADR-based constructs have been explored for in vivo targeted cancer therapy, suggesting that CellREADR holds promise for in vivo applications [19,51,52]. Nevertheless, several limitations of the current system merit careful consideration. In our tandem reporter–effector configuration, the effector (copGFP) signal was consistently attenuated relative to the reporter (mCherry) across all conditions examined. This disparity may pose a particular challenge in vivo, where tissue autofluorescence and elevated background noise could compromise the reliable detection of the comparatively weak effector signal. Additional hurdles include the efficient delivery of sesRNA constructs to target tissues, the long-term stability of sensor expression under physiological conditions, and the potential for immunogenicity. Therefore, while our findings—considered alongside the growing body of in vivo evidence from other systems—support the translational potential of CellREADR, further optimization of the expression architecture and rigorous validation in appropriate in vivo models are prerequisite to its routine application in lineage tracing.

Finally, the future optimizations of this promising system include the following: First, sensitivity enhancement by more careful selection of marker genes and more rational design of sesRNAs to endure higher level expression of sesRNAs with reduced RNAi. Second, more accurate cell type/state identification by using a combination of multiple marker genes. Third, better acid delivery methods such as the use of lipid nano-particles for tough cells such as mSSCs so that the plasmids or in vitro transcribed sesRNAs can be delivered into cells more efficiently.

## Data Availability

The original contributions presented in this study are included in the article. Further inquiries can be directed to the corresponding author.

## Supplementary Materials

The following supporting information can be downloaded at: https://www.mdpi.com/article/10.3390/biotech15040077/s1. Supplementary Figure S1. Gating strategy used for flow cytometry analysis; Supplementary Figure S2. Gating strategy for sorting and primer validation; Supplementary Figure S3. Results of flow cell analysis and mutation detection; Supplementary Figure S4. STO-GDNF cell line establishment and sequences comparison; Supplementary Figure S5. Exploring the accuracy of CellREADR in similar sequences detection; Supplementary Figure S6. Flow cytometric analysis and fluorescence imaging after 2 months of culture of ses-PLZF-W-labeled mSSCs; Supplementary Figure S7. The original image of Western blot used in Figure 4d and Figure 5a; Supplementary Table S1. Six genes that are differentially expressed in HEK293 and HeLa cells, data from Jin, H, et.al [40]; Supplementary Table S2. The list of qPCR primers used in this study. “q-m” indicates the primer designed for mouse, “q-hu” indicates the primer designed for human; Supplementary Note S1. sesRNA sequences used in this study.

## Abbreviations

ADAR	Adenosine deaminase acting on RNA
CellREADR	Cell access through RNA sensing by Endogenous ADAR
mSSCs	Mouse spermatogonial stem cells
mESCs	Mouse embryonic stem cells
RA	Retinoic acid
WPRE	Woodchuck Hepatitis Virus Posttranscriptional Regulatory Element
GOI	Gene of interest
MEF	Mouse embryonic fibroblast
mRNPs	Messenger ribonucleoproteins
