# Supplementary material for: Long-Term Labeling and Differentiation Monitoring of Mouse Spermatogonial Stem Cells Using CellREADR
Source: BioTech (Basel). 2026 Sep 8;15(4):77. doi: 10.3390/biotech15040077 (PMC13600034; doi:10.3390/biotech15040077)
Supplement: Supplementary file 1 [file biotech-15-00077-s001.zip › biotech-4465085-supplementary.pdf]

BioTech

MDPI

Supplementary Materials: Long-Term Labeling and Differentiation Monitoring of Mouse Spermatogonial Stem Cells using CellREADR

Shuaitao Hu, Lin Yan, Gulistan Khan, Xiaowei Liu and Chunsheng Han*

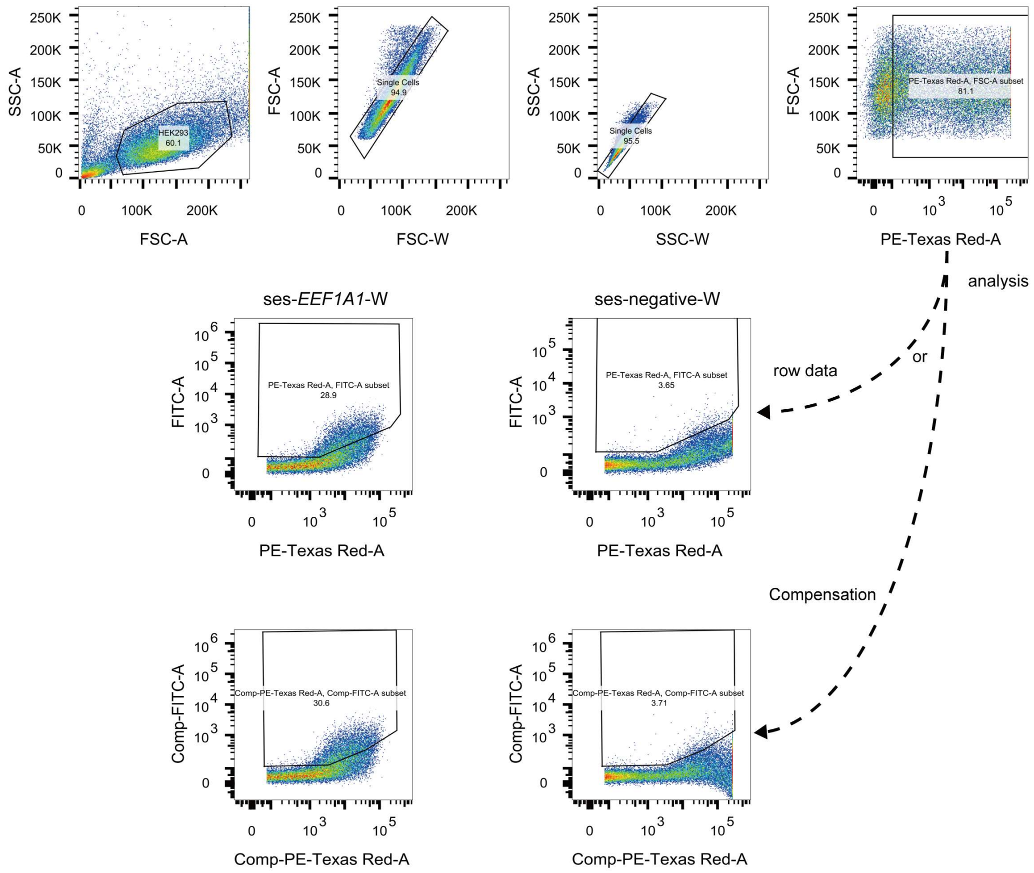

Supplementary Figure S1. Gating strategy used for flow cytometry analysis. Taking HEK293FT cells as example, gates are drawn using a control population transfected with ses-negative-W. Except for gating in Fig. 4c in HeLa, row data was directly analyzed without compensation.

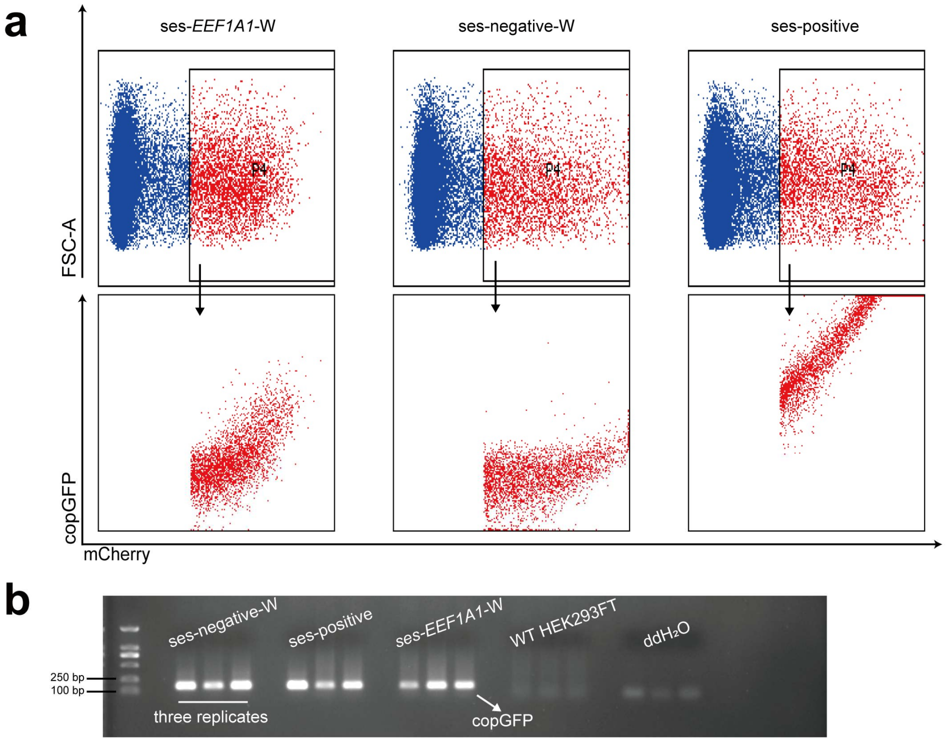

Supplementary Figure S2. Gating strategy for sorting and primer validation. (a) Gating strategy for sorting strong mCherry-positive cells in P4 region; the correspondent copGFP intensity of those cells are shown below. (b) Validating primers targeting copGFP via RT-PCR using RNA from sorted mCherry-positive cells (ses-negative-W, ses-positive, and ses-*EEF1A1*-W groups), with RNA from WT HEK293FT cells and ddH2O as negative controls.

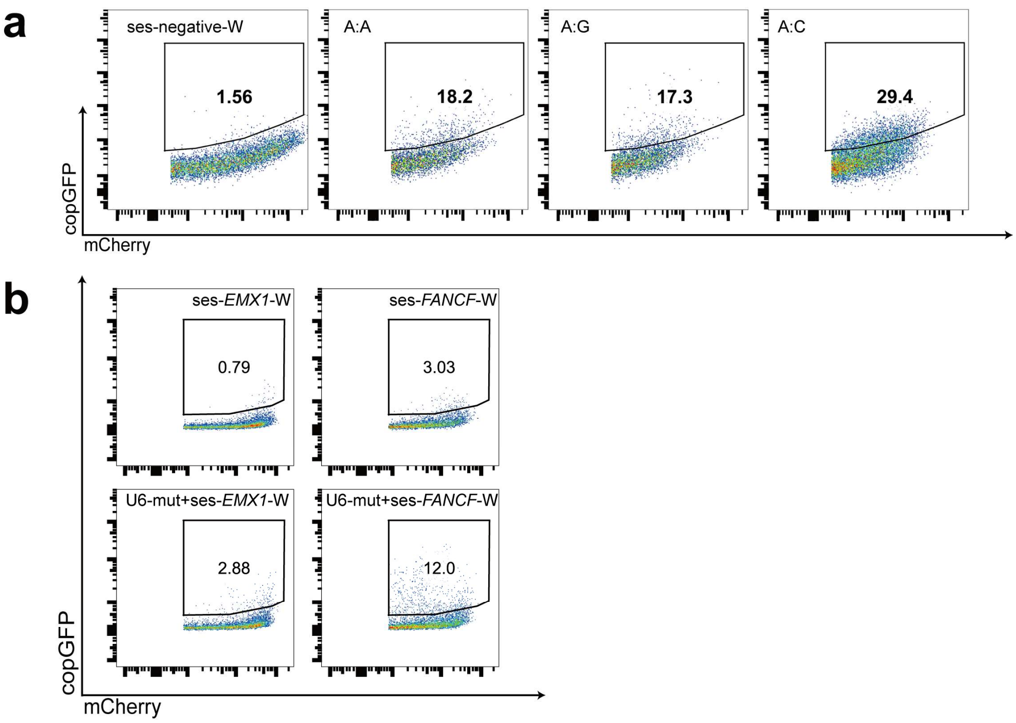

Supplementary Figure S3. Results of flow cell analysis and mutation detection. (a) Gating results on ses-negative-W, A:A mismatch, A:G mismatch and A:C mismatch. (b) Gating results on mutation detection.

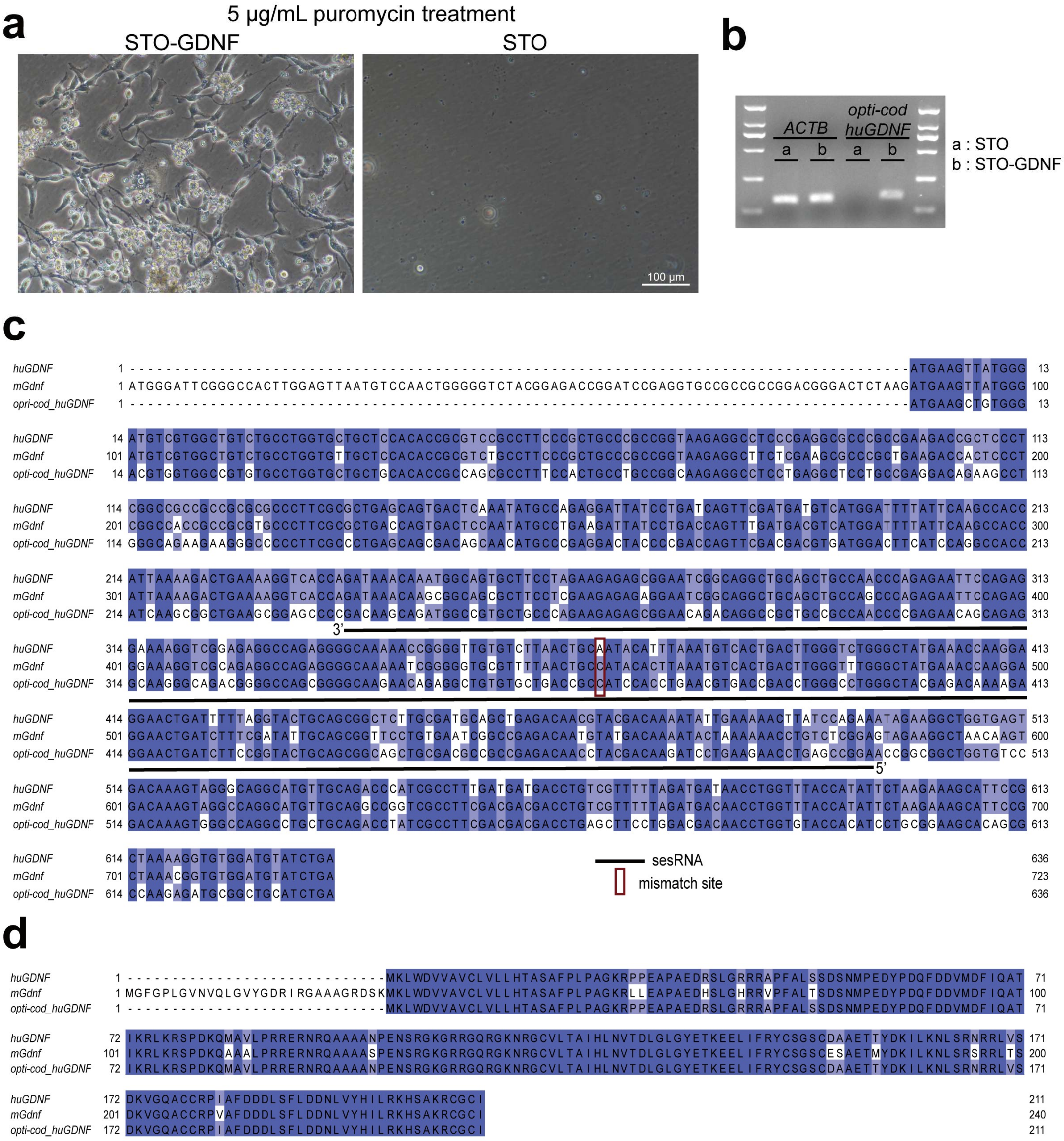

Supplementary Figure S4. STO-GDNF cell line establishment and sequences comparison. (a) The remaining cell under 5 µg/mL puromycin treatment is STO-GDNF cell line, while the WT STO cell is the parallel control all dead under treatment; scale bar is 100 µm. (b) Genotyping STO-GDNF cell using PCR by targeting *opti-cod huGDNF* sequence. (c) Multiple coding sequence alignment of *mGdnf*, *huGDNF* and *opti-cod huGDNF*. (d) Multiple protein sequence alignment of mGDNF, huGDNF and opti-cod huGDNF.

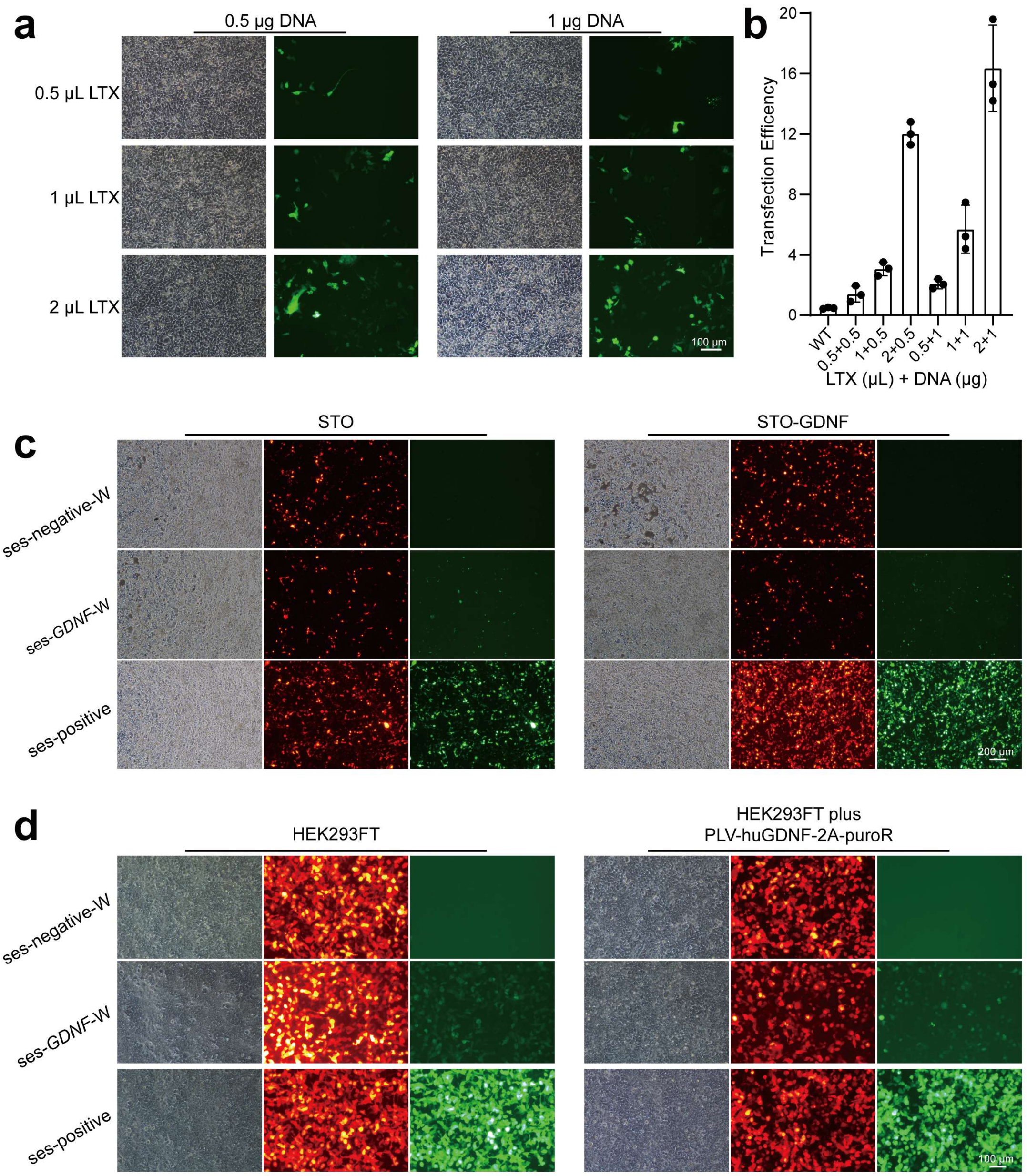

Supplementary Figure S5. Exploring the accuracy of CellREADR in similar sequences detection. (a) To find out the suitable transfection system in STO cell, the LTX reagent was set into three gradients: 0.5 μL, 1 μL, 2 μL. The plasmid DNA was set into two gradients: 0.5 μg, 1 μg. Scale bar is 100 μm. (b) Statistic results of transfection efficiency, n = 3. (c) Florescent images in ses-negative-W, ses-*GDNF*-W and ses-positive groups between STO and STO-GDNF cell lines; scale bar is 200 μm. (d) Fluorescent images in ses-negative-W, ses-*GDNF*-W and ses-positive in HEK293FT supplement with or without PLV-huGDNF-2A-puroR; scale bar is 100 μm.

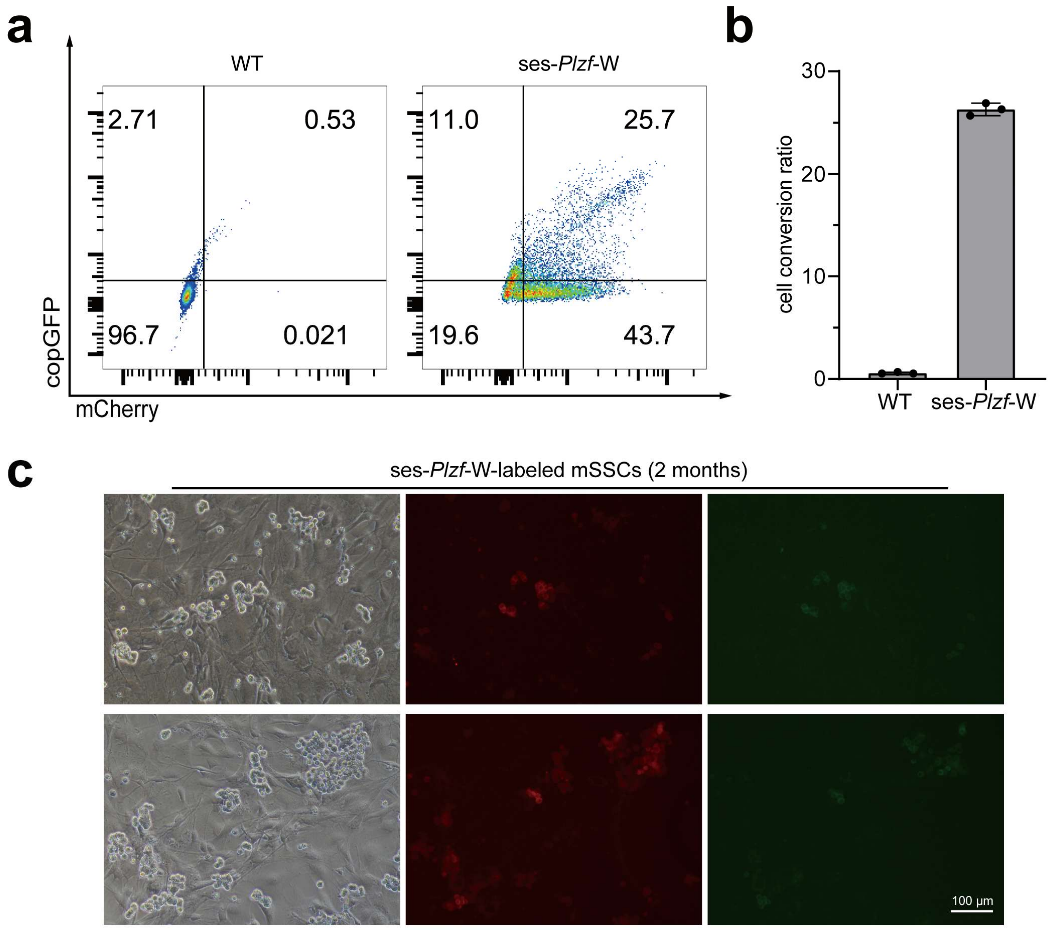

Supplementary Figure S6. Flow cytometric analysis and fluorescence imaging after 2 months of culture of ses-PLZF-W-labeled mSSCs. (a) Flow cytometry results for WT and ses-*Plzf*-W groups. (b) Statistical results of the cell conversion ratio. (c) Fluorescence images on 2 months of the labeled mSSCs.

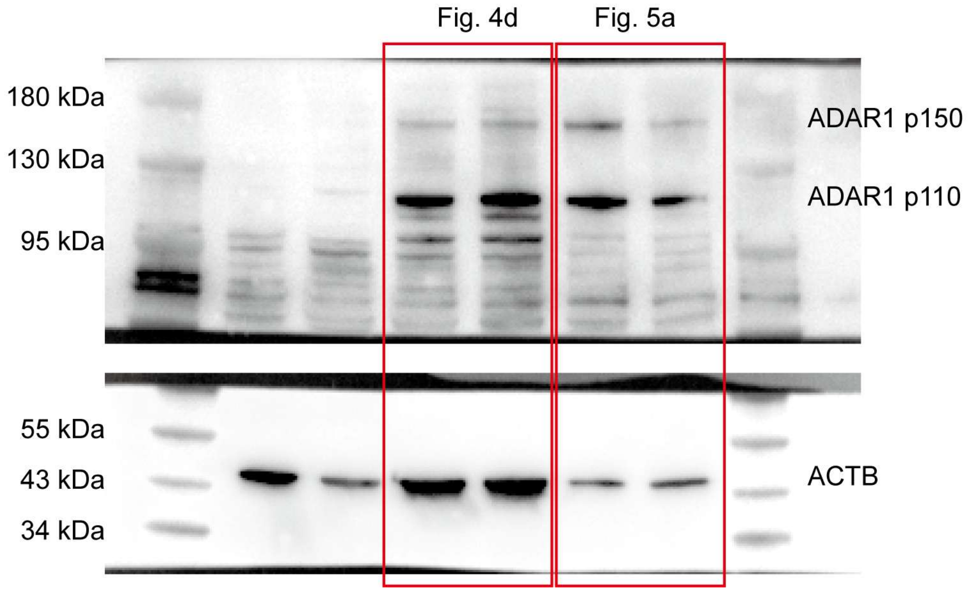

Supplementary Figure S7. The original image of Western blot used in Fig. 4d and Fig. 5a.

Supplementary Table S1. Six genes that are differentially expressed in HEK293 and HeLa cells; data from Jin, H, et.al [40].

Gene	FPKM in cell types				Gene.type	Description
	HEK293 repeat-1	HEK293 repeat-2	HeLa repeat-1	HeLa repeat-2		
NEFM	3392.16	3529.00	0.00	0.00	protein_coding	neurofilament medium chain
LCP1	177.21	183.82	0.70	0.00	protein_coding	lymphocyte cytosolic protein 1
CSNK2B	295.80	277.52	0.58	0.29	protein_coding	casein kinase 2 beta
SPINK6	0.00	0.00	4336.21	1009.85	protein_coding	serine peptidase inhibitor Kazal type 6
KRT7	0.00	0.00	1305.11	367.58	protein_coding	keratin 7
PDE2A	0.04	0.31	421.44	251.03	protein_coding	phosphodiesterase 2A

Supplementary Table S2. The list of qPCR primers used in this study. "q-m" indicates the primer designed for mouse, "q-hu" indicates the primer designed for human.

**q-huACTB-F**	**CATGTACGTTGCTATCCAGGC**
q-huACTB-R	CTCCTTAATGTCACGCACGAT
q-mACTB-F	GTGACGTTGACATCCGTAAAGA
q-mACTB-R	GCCGGACTCATCGTACTCC
q-copGFP-F	GATGGGCTACGGCTTCTACC
q-copGFP-R	CCTCGTAGCGGTAGCTGAAG
q-huEMX1-F	ACCGGGACCCTCTCCATTT
q-huEMX1-R	GCTTCTGCCGTTTGTACTTTGT
q-huFANCF-F	AACCCGGCATCCACAAATCA
q-huFANCF-R	GTGGCGGCTAGTCACTAAAGT
q-huKRT7-F	TCCGCGAGGTCACCATTAAC
q-huKRT7-R	GCTCTGTCAACTCCGTCTCAT
q-huLCP1-F	GATCAGTGTCCGATGAGGAAATG
q-huLCP1-R	CCAGATCACCTGTAGCCATCA
q-huSPINK6-F	TGACTGTGGTGAGTTCCAGGA
q-huSPINK6-R	CCACTTTTCACTATGGCCTTACA
q-huNEFM-F	GAAATCGCTGCGTACAGAAAAC
q-huNEFM-R	TAATGGCTGTCAGGGCCTCTT
q-huPDE2A-F	GACCGCAAGATCCTCCAACTG
q-huPDE2A-R	CCGAGCACTTTGTCTCCGA
q-huCSNK2B-F	GCTCTTCATGGTGCATCCCG
q-huCSNK2B-R	GCGAATCGTCTTGACTGGGC
q-huGDNF-F	GGCAGTGCTTCCTAGAAGAGA
q-huGDNF-R	AAGACACAACCCCGGTTTTTG
q-mGDNF-F	AGAGGGGCAAAAATCGGGG
q-mGDNF-R	CCGCTGCAATATCGAAAGATCA
q-huADAR-F	ATCAGCGGGCTGTTAGAATATG
q-huADAR-R	AAACTCTCGGCCATTGATGAC
q-mADAR-F	CACGGTGTTCACCCTACAAGA
q-mADAR-R	CGAGGTTCATGGGACGGTC
q-huADAR2-F	CGCGCCTTTGTTTGTCATGTC
q-huADAR2-R	GGAAACTGAACGAAAGACCTCAA
q-mADAR2-F	GCGAGGGTATTCCGCTCTC
q-mADAR2-R	GGTACTTGGAGTGACCATTGCT
q-mPLZF-F	CTGGGACTTTGTGCGATGTG
q-mPLZF-R	CGGTGGAAGAGGATCTCAAACA

Supplementary Note S1. sesRNA sequences used in this study

The word in red is functional stop codon or artificial mutation.
The word in green is complementary to the sesRNA sequence.

Ses-negative (a short linker):

GGATCCTAATAGGCTAGC

Ses-positive (a short linker):

GGATCCGGCGCTAGC

ses-EEF1A1 (A:C mismatch):

GCCATCTTCCAGCTTTTTACCAGAAC-GGCGATCAATCTTTTCCTTCAGCTCAGCAAACTT-GCATGCAATGTGAGCCGTGTGGCAATCCAATACAGGGGCATAGCCGGCGCTTATTTGGCCTAGATGGTTCAGGATAATCACCTGAGCAGTGAA-GCCAGCTGCTTCCATT-GGTGGGTCATTTTTGCTGTCACCAGCAACGTTGCCACGACGAACATCCTTGACAGACACATTCTTGACATTGAAGCCCAC

ses-EEF1A1 (A:A mismatch):

GCAGTGAAGCCAGCTGCTTCCATTGGTGGGTCATTTTTGCTGTCACCAG-CAACGTTGCCAC-GACGAACATCCTTGACAGACACATTCTTGACATTGAAGCCCACATAGTCCCCAGGAAGAGCTTCACTCAAAGCTTCATGGTGCATTTCGACAGATTTTACTTCCGTT-GTAACGTT-GACTGGAGCAAAGGTGACCACCATACCGGGTTTGAGAACACCAGTCTCCACTCGGCCAACAGGAACAGTACCAATACCACCAATTTTG

ses-EEF1A1 (A:G mismatch):

GCAGTGAAGCCAGCTGCTTCCATTGGTGGGTCATTTTTGCTGTCACCAG-CAACGTTGCCAC-GACGAACATCCTTGACAGACACATTCTTGACATTGAAGCCCACATTGTCCCCAGGAAGAGCTTCACTCAAAGCTTCATGGTGCATTTAGACAGATTTTACTTCCGTT-GTAACGTT-GACTGGAGCAAAGGTGACCACCATACCGGGTTTGAGAACACCAGTCTCCACTCGGCCAACAGGAACAGTACCAATACCACCAATTTTG

ses-EEF1A1 (TGA as functional stop codon):

GCCATCTTCCAGCTTTTTACCAGAAC-GGCGATCAATCTTTTCCTTCAGCTCAGCAAACTT-GCATGCAATGTGAGCCGTGTGGCAATCCAATACAGGGGCATAGCCGGCGCTTATTTGGCCTGAATGGTTCAGGATAATCACCTGAGCAGTGAA-GCCAGCTGCTTCCATT-GGTGGGTCATTTTTGCTGTCACCAGCAACGTTGCCACGACGAACATCCTTGACAGACACATTCTTGACATTGAAGCCCAC

ses-EMX1:

ACAGCCCCGCGCGCCCCCGAGCCTCCCCCGCATCGCCGCAGCAC-GGGCGCCGGCCGGCCGCCTCGGCGGGCACAGCGTGGACACTCACCCTGGAAGCGGTGGCCGAAGAAGCGGTTCCGCAGGACCCAGGGGTAGAAATGGA-GAGGGTCCCGGTGCTGGGCGCCGAAGAAGGAGTGCGGGGGCTGCAGCGGGGAGGCGCCCAGCTGGTGCGCCGGATGCACGGTCAGCGCGGGGTGGTTC

U6-EMX1-mut:

GCGCTCAACTACCCTCACCCCAGCGCGGCCGAGGCGGCCTTCGTGAG-TGGCTTCCCTGCCGCGGCCGCCGCGGGCGCGGGCCGCTCGCTCTACGGTGGGCCCGAGCTCGTGTTCCCCGAGGCCATGAACCACCCCGCGCTGACCGTG-CATCCGGCGCAC-CAGCTGGGCGCCTCCCCGCTGCAGCCCCCGCACTCCTTCTTCGGCGCCCAGCACCGGGACCCTCTCCATTTCCACCCCTGGGTCCTGCGGAACCGCTTCTTCGGCCAC-CGCTTCCAGGGTGAGTGTCCACGCTGTGCCCGCCGAGGCGGCCGGCCGGCGCCC

GTGCTGCGGCGATGCGGGGGAGGCTCGGGGGCGCGCGGGGCTGTTA-
GAAGTTACTGCCGG-
GAAGGCTGCAGGTCCGCGGAGGTAGATTCCCAGGCAGGGAAGAGCTGTGCGGC
ATCCACCCGCGCCTTCGCCGCGTAGGTCTCCCTCCCAGGAAAGCAGGTGGA-
GACCTCCAGGC

ses-FANCF:

CCGAGGGCCTGGAAGTTCGCTAATCCCGGAACTGGACCCCGCCCAAA-
GCCGCCCTCTT-
GCCTCCACTGGTTGTGCAGCCGCCGCTCCAGAGCCGTGCGAATGGGGCCATGCC
GACCAAAGCGCCGATGGATGTGGCGCAGGTAGCGCGCCCACTG-
CAAGGCCCGGCGCAC-
GGTGGCGGGGTCCCAGGTGCTGACGGAGGTAGTGCTTGAGACCGCCAGAAGCTC
GGAAAAGCGATCCAGGTGCTGCAGAAGGGATTCC

U6-FANCF-mut:

ACACGGATAAAGACGCTGGGAGATTGACATGCATTTCGACCAATAGCATT-
GCAGAGAGGCG-
TATCATTTCGCGGATGTTCCAATCAGTACGCAGAGAGTCGCCGTCTCCAAGGTG
AAAGCGGAAGTAGGGCCTTCGCGCACCTCATGGAATCCCTTCTGCAGCACCTG-
GATCGCTTTTCCGAGCTTCTGGCGGTCTCAAGCACTACCTACGTCAGCACCTGGG
ACCCCGCCACCGTGCGCCGGGCCTTGCAGTGGGCGCGCCAC-
CTGCGCCACATCCATCGGCGCTTTGGTCGGCATGGCCCCATTCGCACGGCTCTGG
AGCGGCGGCTGCACAACCAGTGGAGGCAAGAGGGCGGCTTT-
GGGCGGGGTCCAGTTCCGG-
GATTAGCGAACTTCCAGGCCCTCGGTCACTGTGACGTCCTGCTCTCTCTGCGCCT
GCTGGAGAACCGGGCCCTCGGGGATGCAGCTCGTTACCACCTGGTGCAG-
CAACTCTTTCCCGGCCCGGGCGTCCGGGACGCCGATGAGGAGACACTCCAAGA
GAGCCTGGCCCGCCTTGCCCGCCGGCGGTCTGCGGTG-
CACATGCTGCGCTTCAATGGCTATA

ses-KRT7:

ATGAGTTCCTGGTACTCACGCAGCTGCCGTGCCATATCCTGCTT-
GGCCCGCTG-
CAGGGCGGCTTCCAGCTCCTCCTGCTTGGCACGAGCATCCTTGAGCGCCAGCTCC
CCACGCTCCTCAGCCTCGGCAATGGCGGCCTCCAACTTGGCACGCTAGTTCTT-
GATGTT-
GTCGATCTCAGCCTGCAGCCTCTGGATGGCCCGGTTCATCTCTGAAATCTCATTC
CGGGTATTCCGGAGGTCGTCCCCATGCTTCCCAGCCTGGGCCTG-
GAGGGTCTCAAAC

ses-NEFM:

CCTTCAGCTTCAGCTTCTGTTTCTCCTTCTTCCTGCTCAC-
CTTCCTCTTTTTCACTA-
GAGCCTTCCTTCTCGGATCCTCCCTCTTCGGCTTGGTCTGACTTAGCTCCCTCATC
TTCTTCCTCCTCTTCTTCCTAGCCTTCTTCTTCCTCCTTTTCCCCTTCCTCTTCTTTAA
CTTCAGGTGCAGTTGCTTTCACTGGAGACTTTTTGGCAGC-
TACTTCTTCTTCTTCAGCTTCGGGTTCCTCTTCCTTTTCTTCTGCTGCTTCT

ses-Klf4:

GGACCGGCTGACCGTGCAGGACGGGACCGCCTCTTGCTTAATCTTGGGG-
CACATGCGCGGCGGGCCACCGCTGTAGGGCGCCACTACCACGGGGTGGCTGCC
GTCTAGGCTTCCTTTGCTAACACTGATGACCGAAGGGCTGCTG-
TACTCGCTGCCAGGGGTGGTCAGAGACGCCTTCAGCACAAACTTGCCCATCAGC
CCGCCACCTGGCGGCTGAGGCTGCTGTGGCG-
GAATGTATACTGGGTCCAACTCCGGCCGCAGGAGCTCAGCCACGAAGCC

ses-Plzf:

AGCGTTTCCCACACAGCAGACAGAAGACAGCCATGTCCGTGCCAG-
TATGGGTCTGTCTGTGTGTCTCCAGGGCATCCTCCTTTGAGAACTGGGCACCGCA
TTGATCACACACAAAGGCTTTAGCACCCGCTGAATGAGCCAG-
TAAATGCATTCTCAGTCG-
CAAACTGTCCAGGAACCGTTTTCCGCAGAGTTCACACCCGTATGTTTTCATCCCA
CTGTGCAGTTTCCTGTGCTGCTCCACTGCCTCGTTGTCCG
